# Characterizing phenotypic diversity in marine populations of the threespine stickleback

**DOI:** 10.1038/s41598-022-22872-z

**Published:** 2022-10-26

**Authors:** Ainsley Lilias Fraser, Rana El-Sabaawi

**Affiliations:** Department of Biology, University of Victoria, Victoria, BC Canada

**Keywords:** Ecology, Evolution

## Abstract

The threespine stickleback (*Gasterosteus aculeatus*) is an important model for studying the evolution of vertebrate morphology. Sticklebacks inhabit freshwater, brackish, and marine northern hemisphere waters. Anadromous and marine populations (hereafter marine) are assumed to have remained unchanged morphologically from ancestral marine sticklebacks, despite marine environments varying on regional and local scales. Recent studies suggest that genetic and phenotypic structure exists in marine populations, yet the scale of this variation, and its ecological causes remain unclear. Our goal was to assess morphological trait variation in marine stickleback populations around Southern British Columbia (BC) and determine if oceanographic and habitat characteristics were associated with this variation. Between May–July 2019, we sampled 534 sticklebacks from 15 sites around Vancouver Island, a region characterized by a large diversity of oceanographic and habitat features. We characterized trait variation using two-dimensional (2D) geometric morphometric analysis, comparing individuals between oceanographic regions and habitats. We focused on head and body shape. We found that marine sticklebacks varied morphologically among and between regions and habitats, but the variation did not appear to be related to environmental variation. Sexual dimorphism was the largest source of variation, but oceanographic and habitat variables influenced differences between sexes. We concluded that marine sticklebacks offer abundant opportunities for expanding our knowledge of drivers of morphology.

## Introduction

The threespine stickleback (*Gasterosteus aculeatus*) is one of the most important models for studying the evolution of vertebrate morphology. Marine, anadromous, and freshwater stickleback populations are widely distributed across temperate and boreal regions in the northern hemisphere^1^. The presence of so many distinct stickleback populations gave researchers an excellent opportunity to observe the effects of differing environments on phenotype, life history, and behaviour^2–9^. To date, most large-scale studies of morphological variation in sticklebacks examine freshwater populations^10–14^. Consequently, the ecological factors driving morphological evolution in freshwater ecosystems have been well-studied, while relatively little is known about the ecological factors structuring marine populations^15–18^.

Because marine sticklebacks can potentially migrate between open water and coastal regions, appear to be unhindered by physical barriers in the coastal environment that might impede gene flow, and possess a relatively unaltered morphology compared to the fossil of a marine stickleback, the idea that marine sticklebacks have remained unchanged over thousands of years has been treated as a fundamental assumption in stickleback research^1,19–21^. Despite occurring in environments that are geographically and oceanographically isolated, different marine populations have been used in studies to represent ancestral stickleback morphology^9,22–28^.

Until now, evidence of local adaptation and small-scale genetic structuring in marine sticklebacks has primarily been characterized in either a single small basin (i.e. the Baltic Sea)^29–31^, or across very large basins (e.g. the Atlantic vs. Pacific Oceans)^32–34^. However, there are currently no studies on a medium scale (100–1000 km), where a variety of oceanographic features and habitat types can be found. The coast of Vancouver Island and the coast of Southern mainland BC are optimal locations to conduct this research because sticklebacks are widely distributed in this region. With a mesothermic maritime climate and one of the longest fjord coastlines in the world, BC has a unique and diverse coastline^35^. This is also where previous studies have found considerable diversity in freshwater lake and stream stickleback populations.

There is considerable environmental variation on medium scales that may have an important structuring effect on marine stickleback populations^36–38^. Factors including temperature and salinity do not vary randomly in the ocean but are governed by underlying oceanographic variation^39^. Many studies have found that sticklebacks have a large capacity for phenotypic plasticity in body shape in response to salinity, temperature, and habitat characteristics^18,24,40–48^. However, body shape has not been compared among marine stickleback populations inhabiting high salinity environments, and it is unknown if small variations in salinity could influence body shape variation^22,49^.

Habitat characteristics, such as benthic availability (i.e. average water depth in a habitat) and predator presence, also correlate with morphological variation in freshwater sticklebacks^12,17,50–55^. For example, sticklebacks found in a shallow habitat with higher benthic habitat availability, that feed on large benthic invertebrates, have deep bodies, large heads, and large caudal fins. While, sticklebacks from deeper, limnetic habitats, that feed on mobile zooplankton in the water column, generally have elongated, shallow body shapes, small heads, and smaller caudal fins^12,56^. However, it is currently unknown whether marine sticklebacks vary in response to these environmental features, or if the ecological factors driving morphological variation in freshwater sticklebacks also drive variation in marine sticklebacks.

Additionally, sticklebacks are sexually dimorphic in both behavioural and morphological traits, and is often one of the largest sources of intraspecific variation in this species^57^. In marine populations specifically, females are larger than males and males have larger heads than females, as seen in freshwater populations^6,44,58,59^. Characterizing sexual dimorphism allows us to assess how variation relating to oceanographic and ecological factors compares to variation relating to sexual dimorphism, providing context for the relative importance of these factors.

This study broadly aims to assess morphological variation among marine sticklebacks across the medium spatial scale of Vancouver Island and Southern mainland BC and has two main objectives. The first objective is to characterize morphological variation among and within these populations: between sexes, and among regions and habitats. The second objective is to assess whether this variation can be attributed to specific physical factors (i.e. oceanographic regime, habitat characteristics). We examined two main aspects of morphological variation, head shape and body shape.

Our sampling area covered distinct oceanographic regions around Southern BC: The Strait of Georgia, the Juan de Fuca Strait (both part of the Salish Sea), the northern coast of Vancouver Island, and the west coast of Vancouver Island (Supplementary Fig. S1). These four regions are dominated by different oceanographic forces. The Strait of Georgia is estuarine dominated^35^. The Juan de Fuca Strait is dominated by tidal mixing^60^. The west coast of Vancouver Island is influenced by upwelling and multiple fjords providing freshwater runoff^37^. Lastly, the northern coast is a transition zone between the upwelling and downwelling domains of the eastern Pacific^61^. Data from seven BC lighthouses in May, June, and July between 1956–2019 (Supplementary Table S1, Fig. S2), demonstrate that average sea-surface temperatures are highest in the Strait of Georgia and lowest along the northern coast of Vancouver Island^62^. For average salinity, the Strait of Georgia is the least saline and the northern coast of Vancouver Island is more saline. The Juan de Fuca Strait and west coast recorded intermediate temperature and salinity averages^62^. However, departures from this trend at individual sites can be caused by local variation in tidal cycles, freshwater inputs, topography, or urban development. We sampled temperature and salinity at each site in order to assess how local conditions compared to the broader trends observed in the BC lighthouse data.

In addition to large-scale differences in oceanography, the coastal habitats where sticklebacks breed vary in their physical, chemical, and biological structures^63^. Along the Southern coast of BC, marine sticklebacks have been noted in different types of habitats including tidal flats, salt marshes, and lagoons. Most of our sites were in estuaries, except for three: Oyster Lagoon, Salt Lagoon, and Coles Bay, Saanich (Supplementary Table S2). The first and second are coastal lagoons. The third is a tidal flat with eelgrass beds. Inhabiting different habitats could potentially lead to morphological variation in stickleback because of biological and physical difference between habitats. The presence of tidal currents and the proximity to fresh water determine variation of biological and chemical properties in coastal habitats^64,65^.

Tidal flats are exposed to more wave action and have greater pelagic habitat availability than other habitats^66^. In estuaries, tidal flats border salt marshes at the lower intertidal zone^67^. Tidal flats that do not border estuaries are locally affected by small tidal creeks or by tidal processes. Primary productivity is high on tidal flats with eelgrass beds, which in turn provides prey for fish species which feed throughout the water column^68^. Salt marshes are found between the mean sea level of a region and mean highest high water mark^69^. They are semi-enclosed coastal habitats with organic substrate and are connected to the open ocean by a network of tidal channels which carry water and sediment during daily tidal events. Salt marshes are also very productive ecosystems due to the presence of vegetation which provides detritus for invertebrates, and in turn feeds fish and other vertebrates^70^. Lagoons have restricted access to the neighboring ocean, usually with a small channel which connects a lagoon to the open coast or an estuary^71^. They are shallow, sheltered habitats with organic substrate and increased benthic availability. Green and brown filamentous algae blooms are common in the summer months, with prey availability dominated by deposit-feeding benthic invertebrates^72,73^.

We will examine the following hypotheses: that head and body morphology vary between oceanographic regions (Strait of Georgia, the Juan de Fuca Strait, west coast of Vancouver Island, and north coast of Vancouver Island), either because currents act as a barrier between the regions, or because of variation in temperature or salinity among oceanographic regimes. We also hypothesize that there will be habitat differences in head and body morphology. Coastal habitats in BC include lagoons, salt marshes, and tidal flats. If morphological variation is driven by physical structure, we would expect habitats with greater benthic availability (i.e. greater water depth) to have sticklebacks with large heads and a head shape which suits a benthic-dominant niche. Conversely, we would expect pelagic dominant habitats to have sticklebacks with smaller heads and a head shape to suit a pelagic niche. We expect to see body shape and head shape differ between male and female sticklebacks due to sexual dimorphism and will test whether sexual dimorphism patterns are consistent among regions or habitats.

The two most studied coastal marine stickleback ecotypes are anadromous and marine. Anadromous populations migrate to freshwater habitats as adults to breed in summer months and to raise juveniles in fresh water, while marine populations spend their entire life cycle in full-salinity environments^74,75^. The stickleback populations we sampled are likely from a mixture of anadromous and marine populations. Without tracking migration patterns throughout the year (i.e. using tagging or catch-and-release), it would be difficult to distinguish between the two life histories. Thus, we will henceforth refer to all populations as “marine” throughout the paper.

## Results

### Environmental variation

**Figure 1:**
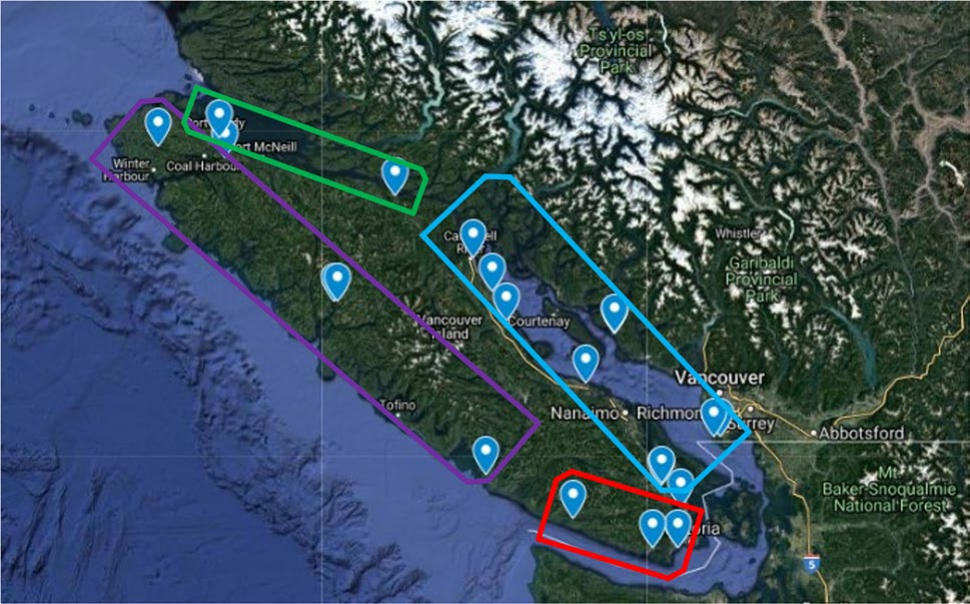
Map of the locations of our sites sampled on the Southern Mainland coast of BC and around Vancouver Island, BC. The Strait of Georgia is in the blue rectangle. The Juan de Fuca Strait is in the yellow rectangle and both Straits make up the Salish Sea. The western coast of Vancouver Island is in the purple, and the northern coast is in the green rectangle. Two points on this map are four separate sites (Moutcha Bay and the site on the Sunshine Coast). The sites were in close proximity on the map, they look like a single point on the map. All latitudinal and longitudinal coordinates can be found above in Table S1. Most sites can be accessed by paved road, but 4 sites were accessed along active logging roads (Bamfield South Inlet, Moutcha Bay (2 Sites), and Holberg, BC). Experience driving on logging roads is crucial. The base map was constructed using Google Earth (open access) at https://earth.google.com/web/.

Despite our sampling sites being further inland (Fig. 1, Supplementary Table S1), there were broad similarities between our data and the BC lighthouse dataset, with a few expectations^62^. The Strait of Georgia (SoG) sites and the SoG lighthouses recorded the warmest average temperatures among the regions. The north coast sites and the north coast lighthouses recorded the highest average salinities. The BC lighthouse dataset also showed Juan de Fuca (JdF) and the north coast lighthouses had very similar average temperatures (Supplementary Table S1). We observed similar, cold temperatures between the JdF and north coast sites (Supplementary Fig. S3a). But by definition, spot sampling captures only a snap shot in time, future studies would benefit from more extensive sampling repeated over a field season.

### Head morphological variation

**Figure 2:**
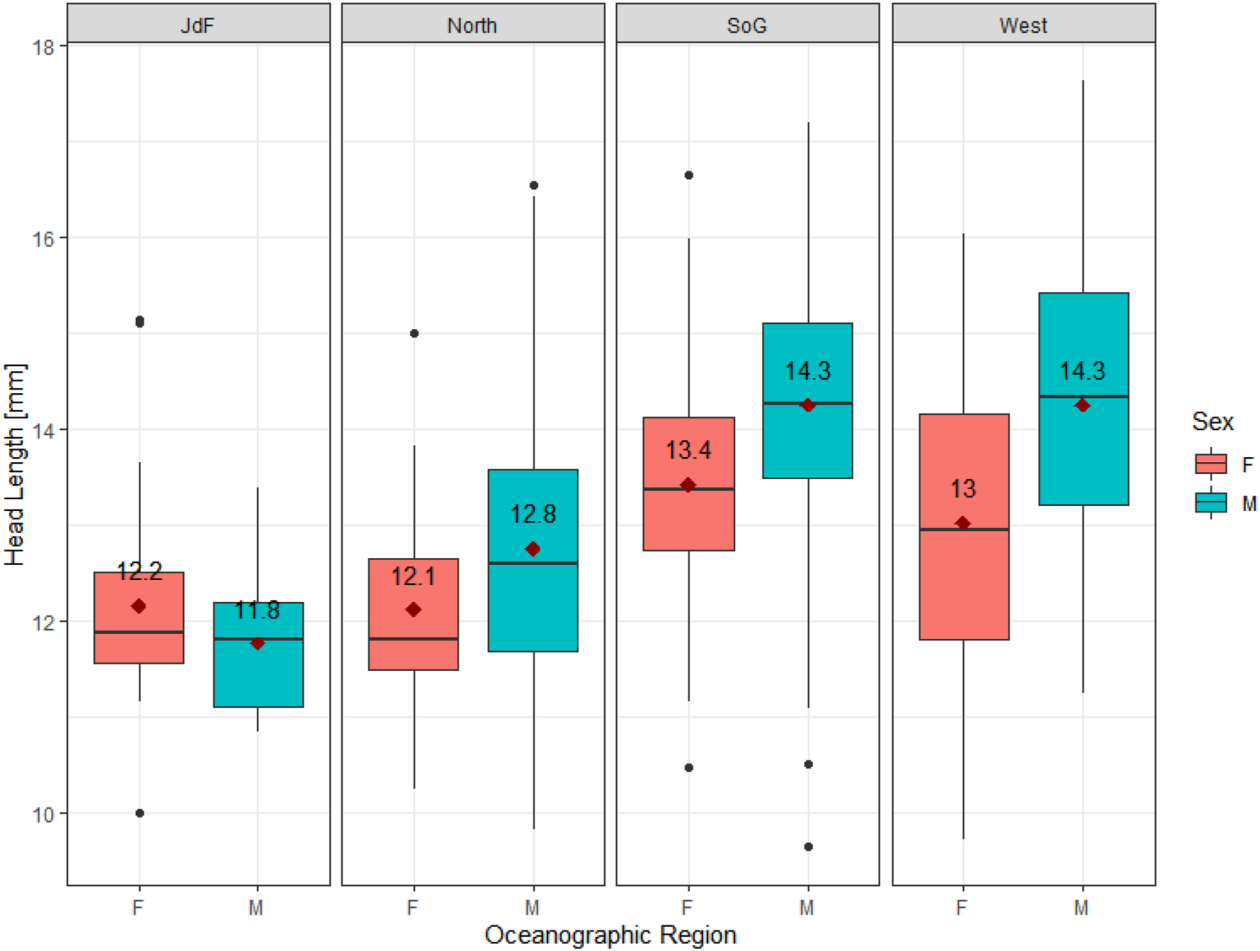
Differences between male (M) and female (F) head size, represented by head length (mm), across oceanographic regions. The regions include the Juan de Fuca Strait (JdF), the northern coast of Vancouver Island (North), Strait of Georgia (SoG), and the west coast of Vancouver Island (West). The solid black lines represent the median of each habitat type, while the red triangles represent average head size for each sex (with the average value in black above each point).

As predicted, there was a significant difference in head size between sexes (*t* = 3.86, *p* =  < 0.001) (Supplementary Table S3). Male head size was, on average, 6% larger in female sticklebacks (Fig. 2) (Welch two-sample *t*-test, *p* < 0.05). There were no significant regional differences in head size (Fig. 1, Supplementary Table S3), but there was also a significant interaction between sex and region (Supplementary Table S3).

A principal components analysis (PCA) on the head shape data, with the centroid size (i.e. CS) excluded, showed that 42.9% of the total head shape variation was explained by the first two Principal Components (PCs)—(PC1 = 24%, PC2 = 18.9%). We constructed a Procrustes linear model (LM), including Log Centroid Size (CS), sex, region, and the interaction of sex and region as fixed factors. Log CS was included because the relationship between head shape and geometric head size was significant (Partial Least Squares correlation coefficient = 0.588, *p* = 0.001, η^2^ = 0.196) (Supplementary Fig. S4). Site was included as a nested random effect.

The regression between head size (CS) and head shape indicated a positive allometry, with a slope of 0.096 for females and 0.1 for males (Supplementary Fig. S5). A regression between head size (CS) and head shape also indicated positive allometry among regions, with a slope of 0.1 at Juan de Fuca sites, 0.11 for the Northern coast sites, 0.14 for the Strait of Georgia and 0.081 for the Western coast (Supplementary Fig. S6). The slopes of the allometry did not vary appreciably between sexes or regions, indicating that it was stable.

**Figure 3:**
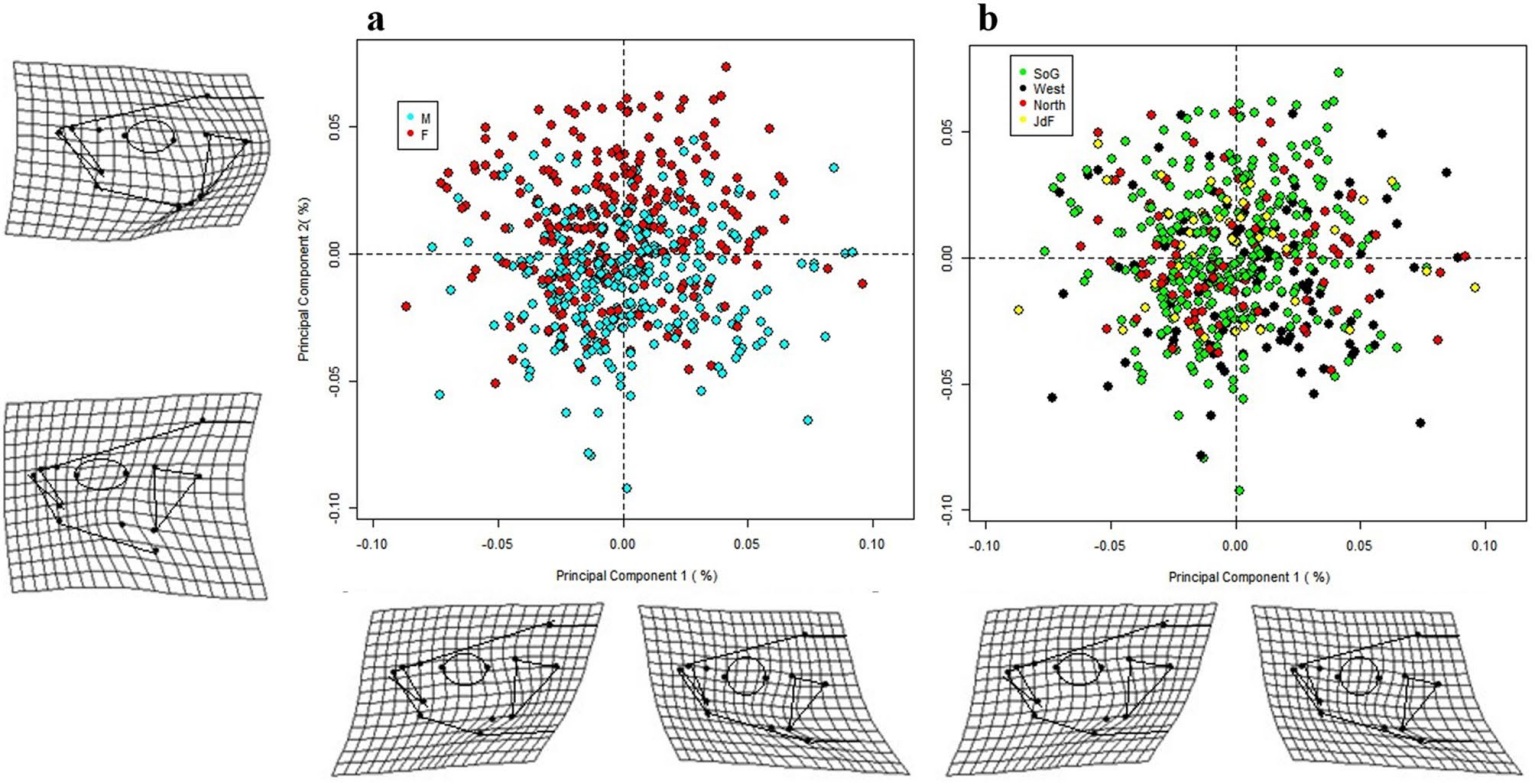
Principal Component Analysis (PCA) of head shape. The head shape for each specimen was obtained from a set of thirteen anatomical landmarks, shown in the methods section (Fig. 6). The landmarks were converted into 26 shape variables using a Generalized Procrustes Analysis (GPA). These variables were used to carry out the PCA. Deformation grids were plotted with 1.5 × magnification to facilitate visualization of head shape differences. The deformation grids represent the difference between the specimen on each end of each shape axis (PC1 and PC2). The effects of body size were removed from this analysis. (**a**) Each data point represents data from a female stickleback (red circles) and male stickleback (light blue circles). (**b**) Each data point represents data from the four oceanographic regions that surround Vancouver Island, BC: Strait of Georgia (green), west coast Vancouver Island (black), north coast Vancouver Island (red), and the Juan de Fuca Strait (yellow).

We found there were significant sex differences in head shape (F_3,534_ = 7.73, *p* = 0.001, η^2^ = 0.139), which accounted for 3.38% of the variation (*R*^2^ = 0.0388) (Supplementary Table S4). However, males and females did not show clear clustering patterns along either PC axis. Instead, there was substantial overlap between sexes (Fig. 3a).

The pattern from the PCA analysis suggested that females had relatively compressed heads with a narrower operculum, while male clustering favored a relatively deeper head and broader operculum (Fig. 3a). This pattern of clustering suggested that males and females were distributed along a continuum between these two head shapes.

There were significant regional differences in head shape (F_1,534_ = 19.01, *p* = 0.001, η^2^ = 0.169), accounting for 3.18% of the variation (*R*^2^ = 0.0318) (Supplementary Table S4). Additionally, there were significant differences between sites nested within regions (F_11,534_ = 6.432, *p* = 0.001, η^2^ = 0.464), accounting for 10.6% of the variation (*R*^2^ = 0.106). However, the PCA plot showed little clustering between regions (Fig. 3b), suggesting a continuum of head shapes along regions rather than stark clustering pattern.

### Body morphological variation

**Figure 4:**
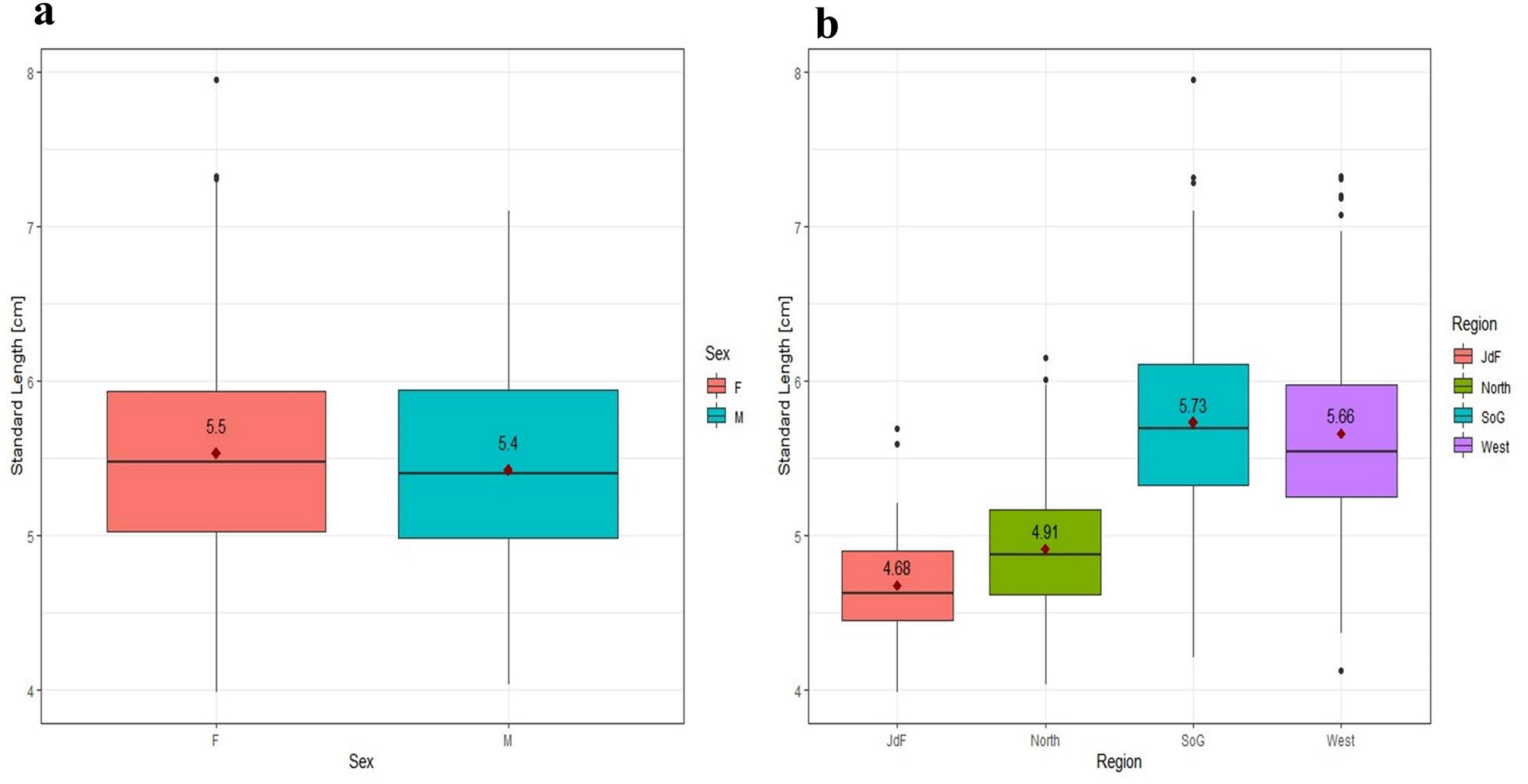
(**a**) Differences between male (M) and female (F) body size, represented by standard length (cm). (**b**) Regional variation between body size observed in four oceanographic regions. The regions include the Juan de Fuca Strait (JdF), the northern coast of Vancouver Island (North), Strait of Georgia (SoG), and the west coast of Vancouver Island (West). The solid black lines represent the median standard lengths. The red triangles represent the average standard length for each sex (with the average value in black above each point).

Body size (i.e. standard lengths) varied between 3.98 cm and 7.95 cm, with an average of 5.48 cm (Table 1). Male and female body size differed significantly (*t* =—3.4, *p* = 0.001) (Supplementary Table S5). As predicted, females had a larger body size than males, average 5.5 cm vs. 5.4 cm (Fig. 4a). There was also significant body size difference among regions (Fig. 4b, Supplementary Table S5). Unlike head size, there was no significant interaction between sex and region, indicating that sexual dimorphism in body size was consistent across regions (Supplementary Table S5, Fig. S7).

**Table 1:** List of sites including each respective site abbreviation, region around Vancouver Island, the habitat type, and the average body size of sticklebacks between males (M) and females (F).

Site Name	Abbreviation	Region	Habitat Type	Average Body Size (cm)	
				M	F
Roberts Bank, off Tsawwassen, BC	RON	Strait of Georgia	Tidal Flat	5.19	6.21
Englishman River Estuary	ENG	Strait of Georgia	Tidal Flat	5.61	6.03
Courtenay River Estuary	COR	Strait of Georgia	Salt Marsh	6.25	6.05
Black Creek Estuary	BCE	Strait of Georgia	Salt Marsh	5.33	5.72
Oyster Lagoon	OYS	Strait of Georgia	Lagoon	5.14	5.49
Coles Bay Regional Park	COL	Strait of Georgia	Tidal Flat	6.31	6.87
Salt Lagoon, Pender Harbour	STL	Strait of Georgia	Lagoon	5.27	5.35
Campbell River, BC	BAK	Strait of Georgia	Salt Marsh	5.45	6.29
Bamfield South Inlet	BAM	Western Coast	Tidal Flat	5.36	5.26
Contuma River Estuary, Moutcha Bay	CMA	Western Coast	Tidal Flat	6.12	6.76
Canton Lagoon, Moutcha Bay	CAN	Western Coast	Lagoon	5.58	5.70
Salmon River, Sayward Estuary	SAY	Northern Coast	Lagoon	4.81	4.87
Holberg Estuary	HOL	Western Coast	Lagoon	4.98	5.42
Port Hardy Estuary	HDY	Northern Coast	Salt Marsh	4.89	5.04
San Juan River	PRF	Juan de Fuca	Salt Marsh	4.35	4.77

**Figure 5:**
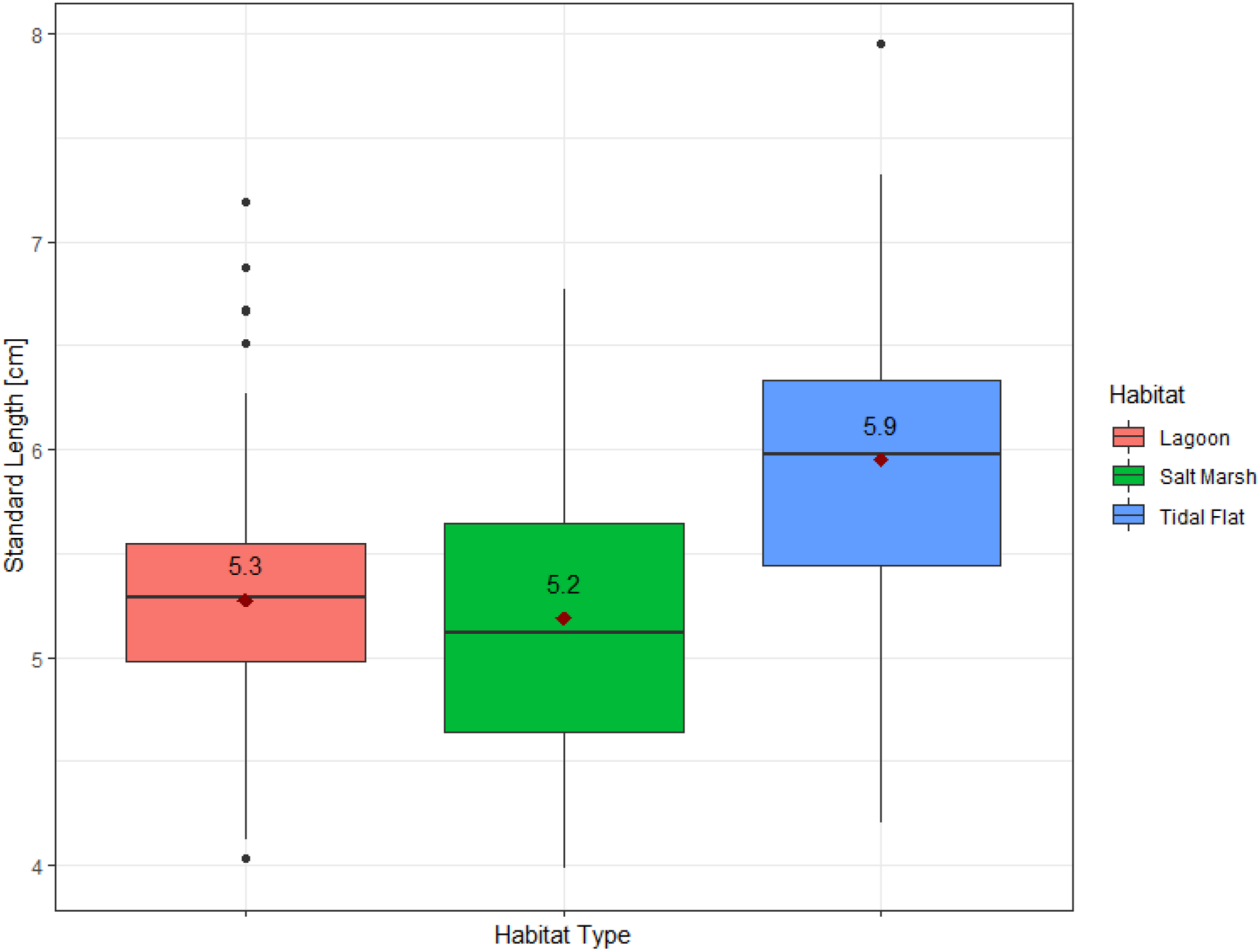
The variation of body size, represented by standard length (cm), observed in each habitat type. The solid black lines represent the median of each habitat. The red triangles represent the average standard length (with the average value in black above each point).

According to the model, body size varied significantly among habitat types (*t* = 2.51, *p* = 0.012 for Tidal Flat level) (Supplementary Table S5). And there was a significant interaction between sex and habitat, indicating that sexual dimorphism in body size depended on the habitat type (Supplementary Table S5). Body size differences were more pronounced in some habitats (tidal flats) than others (lagoons) (Fig. 5). There was also a significant interaction between sex and habitat, indicating that sexual dimorphism in body size depended on the habitat type (Supplementary Fig. S8). Yet, the habitat signatures were quite weak compared to regional variation in body size. (Supplementary Table S5).

A PCA analysis on the body shape data, with centroid size (i.e. CS) excluded, found that 37.6% of the total body shape variation was explained by the first two Principal Components (PCs)—(PC1 = 24%, PC2 = 13.6%). A Procrustes LM for body shape included Log CS, sex, region, habitat, the interaction between sex and region, and the interaction of sex and habitat as the fixed factors. We included Log CS as a fixed factor because the relationship between body shape and the geometric head size was significant (Partial Least Squares correlation coefficient = 0.543, *p* = 0.001, η^2^ = 0.153) (Supplementary Fig. S9).

The regression between body size (CS) and body shape indicated a positive allometry, with a slope of 0.094 for females and 0.043 for males (Supplementary Fig. S10). A regression between body size (CS) and body shape also indicated positive allometry among regions with slopes of 0.051 for the Juan de Fuca sites, 0.052 for the Northern coast sites, 0.073 for the Strait of Georgia and 0.069 for the Western coast (Supplementary Fig. S11). The slopes of the allometry did not vary appreciably between sexes or regions, indicating that it was stable.

**Figure 6:**
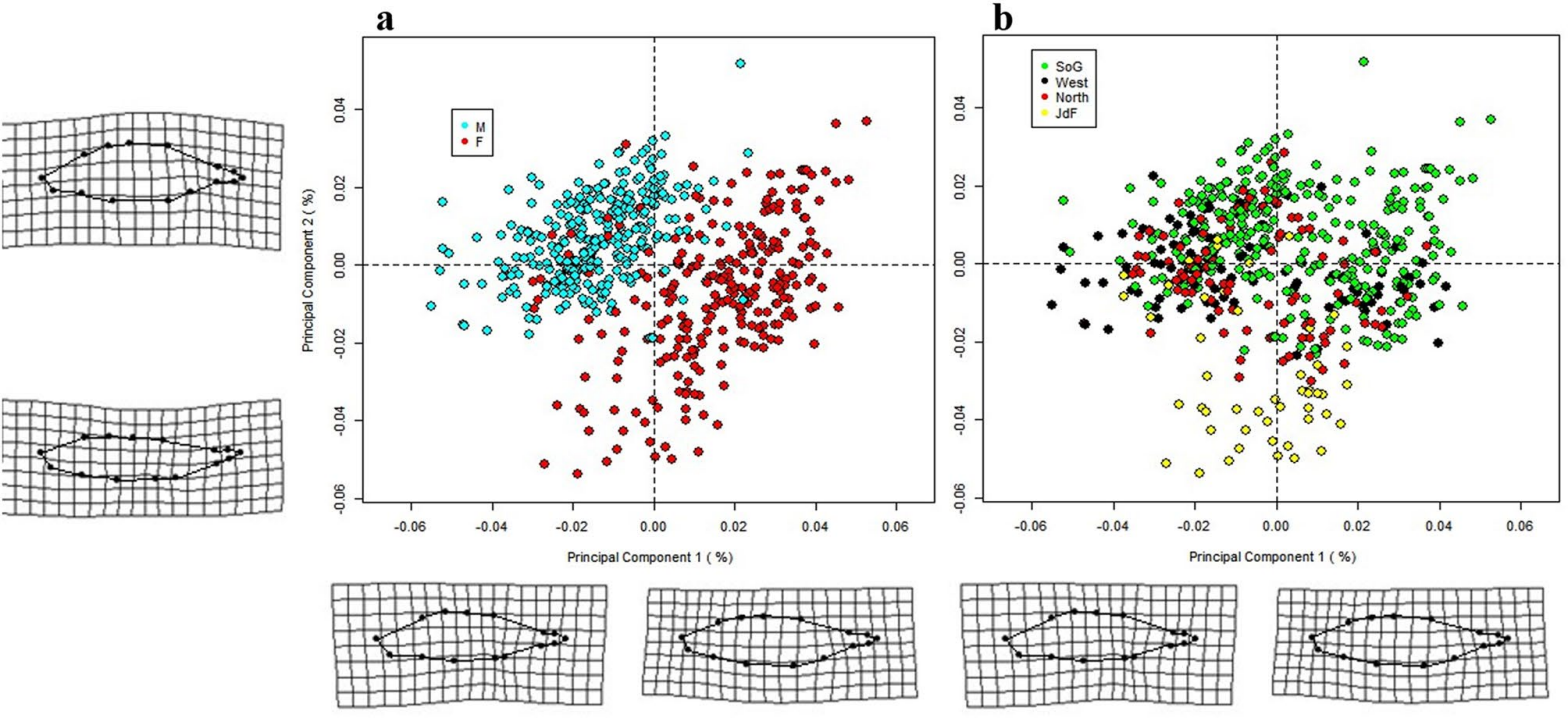
Principal Component Analysis (PCA) of body shape. The body shape for each specimen was obtained from a set of fifteen anatomical landmarks, shown in the methods section (Fig. 7). The landmarks were converted into 30 shape variables by way of a Generalized Procrustes Analysis (GPA). These variables were used to carry out the PCA. The body shape variation is illustrated with the four deformation grids, which represent the difference between the specimen on each end of each shape axis (PC1 and PC2). The effects of body size were removed from this analysis. (**a**) Each data point represents data from a female stickleback (red circles) and male stickleback (light blue circles). (**b**) Each data point represents data from the four oceanographic regions that surround Vancouver Island, BC: Strait of Georgia (green), west coast Vancouver Island (black), north coast Vancouver Island (red), and the Juan de Fuca Strait (yellow).

There was a significant difference between male and female body shapes (*F*_1,534_ = 96.9, *p* = 0.001, η^2^ = 0.390), which accounted for 12.8% of variation (*R*^2^ = 0.128) (Supplementary Table S6). Sticklebacks showed a diffuse clustering pattern by sex along PC1 (Fig. 6a). Female sticklebacks tended to cluster on the right of PC1, towards a deeper body, broader pelvic girdle, a more compressed snout, with larger spacing between dorsal spines. Male sticklebacks tended to cluster toward the left side of PC1, towards a shallower, more streamline body with an elongated snout, smaller relative pelvic girdle, and closely spaced dorsal spines. Female sticklebacks were loosely clustered together, with more variation in body shape, compared to the tightly clustered male sticklebacks (Fig. 6a).

Although there was no noticeable clustering of the body shape data by regions (Fig. 6b), the Procrustes LM showed a significant yet relatively small body shape difference among regions (*F*_3,534_ = 16.8, *p* = 0.001, η^2^ = 0.210), representing 6.66% of the total variation (*R*^2^ = 0.0666) (Supplementary Table S6). The SoG sticklebacks tended to cluster to the left side of PC1 and upper side of PC2 towards a deeper body shape with widely spaced dorsal spines, and a broader pelvic girdle. The JdF sticklebacks clustered towards the bottom of the PC2 axis, or a shallow, more streamlined body shape with compact dorsal spine spacing and a narrower pelvic girdle. As we saw in the body size analysis, the JdF and SoG sticklebacks were the most noticeably distinct regions (Fig. 4b, Supplementary Fig. S7). Additionally, there were significant differences between sites nested within regions (F_9,534_ = 3.50, *p* = 0.001, η^2^ = 0.132), accounting for 42% of the variation (*R*^2^ = 0.420). The location of our sampling sites appears to have a significant effect on body shape variation, but the effect size is smaller than for sex.

### Lateral plate morphology

Of all the fish collected, 97% were complete plate morphs, 2.2% were partial morphs, and 0.56% were low morphs. Each region had non-complete morphs (e.g. Partial and low morphs) present in at least one of the sites (Supplementary Fig. S12). Non-complete morphs were also found in each habitat (Supplementary Fig. S13).

## Discussion

### Overview

The overall goal of this project was to explore morphological variation of marine sticklebacks on the medium spatial scale of Vancouver Island and Southern mainland BC. Our first objective was to explore variation within and among the four oceanographic regions around Vancouver Island. If oceanography on regional scales acted as a barrier between breeding populations, then we predicted stickleback morphology would vary by regions. Our second objective was to explore variation among three coastal habitats where marine sticklebacks are found in BC. Our third objective was to assess morphological differences between males and females, and to compare relative importance of sexual dimorphism to other factors (region and habitat).

### Morphological variation by region

We found variation in head size and shape between regions (Supplementary Table S3, Table S4). However, there was no clear clustering pattern in the PCA plot (Fig. 3b). Instead, head shape varied along a continuum, as opposed to a stark clustering pattern. Morris et al*.* (2018) also found the morphometric shape of six marine stickleback populations from California to BC was distributed along a continuum, although their study analyzed the morphometric variation of the entire body, not the head shape alone^76^. Past research that identified regional phenotypic variation across marine populations primarily analyzed body shape and plate phenotype, but not head morphology^8,30^. There has not been a study, to our knowledge, that examined head morphology of marine sticklebacks and how it is associated with oceanographic regional variation.

Body size and shape varied significantly by region, supporting our hypothesis that we would find variation (Fig. 4b, Supplementary Table S5, Table S6). Some of the variation might be related to temperature differences among the oceanographic regions. Sticklebacks were largest at SoG sites and smallest at the JdF site (Fig. 4b). The temperatures we measured were highest, on average, at SoG sites, and lowest at the JdF site, as well as in the long-term record (Supplementary Table S1, Fig. S3a). Kim et al*.* (2017) found that male sticklebacks bred in environments with higher average winter temperatures were smaller as adults^77^. However, we did not test for a correlation between temperature/salinity and the shape data because our study design used spot sampling, rather than continuous sampling at each site. Therefore our temperature data represent a snap shot in time. It is likely that the regional body shape variation could be described by other drivers, such as factors related to competition for good spawning grounds as Dorgham et al*.* (2018) found in the White Sea marine populations^7^.

### Morphological variation by habitat

Our hypothesis that head shape would vary for a fish that exploits a benthic habitat vs. a fish that is adapted to a pelagic habitat was not supported because we observed no differences in head size or shape among habitats (Supplementary Table S3, S4). This finding agrees with the results of Svanbäck and Schluter (2012), who found that anadromous populations had intermediate head and body morphologies instead of tending toward a benthic or limnetic shape^4^. Future studies should evaluate head morphology according to prey type found at each sampling site, as well as in gut contents, in order to test if varying diets lead to variation in trophic traits in marine sticklebacks^78,79^.

Body size varied among habitats, where sticklebacks were largest on tidal flats, and smallest in salt marsh habitats (Fig. 5, Supplementary Table S5). Seebacher et al*.* (2016) also found that salt marsh sticklebacks in the Great Eau estuary had more slender, less muscular bodies, as well as lower swimming performance relative to other sticklebacks in the estuary^2^. Body shape also varied significantly by habitat, but there was no noticeable clustering in the PCA plot (Supplementary Table S6, Fig. S14). Additionally, the habitat variation did not follow the pattern of benthic-limnetic traits based on habitat availability observed in freshwater stickleback populations^12–15,53–56^. If marine sticklebacks occupy more than one habitat type in their lifetime, their morphologies may be adapted for the variable nature of estuaries as opposed to just one habitat type. The difference in flow speed, for example, could impact body morphology suited for different swimming needs among habitats. Swimming ability is proportional to body size of a fish, larger individuals are able to occupy deeper habitats that have less protection from strong water currents^80–82^. Previous studies have found that the habitat characteristics (i.e. niche and predation pressure) affect key trophic morphological traits such as snout length, orbit size, jaw length, and number of gill rakers^5,16,83–85^. Theses traits were not looked at specifically in our study, as we did not analyze gut contents while dissecting our specimens, but would be helpful to compare between habitat populations in future studies.

Even though we observed that habitats varied in temperature and salinity (Supplementary Fig. S3c, S3d), it did not appear that these variables explain body variation. This contrasts with the findings of DeFaveri and Merilä (2014), who found that salinity had a substantial effect on body size in the Baltic Sea populations^29^. As well as Ramler et al*.* (2014), who found that wild caught marine sticklebacks raised in three different, novel temperatures differed considerably in head and body shape^47^. Therefore, while habitat has a significant impact on stickleback morphology, further investigations are needed to know what elements of a coastal habitat are driving this variation.

### Sexual dimorphism

Head size differed significantly between sexes, which was expected because males generally have larger heads than females^58,86^ (Supplementary Table S3). The interaction between sex and region also had a significant effect on head size, indicating that sexual dimorphism in head size varied among the regions (Fig. 2). Population-specific factors, such as region, have been shown to affect both sexes. Aguirre and Akinpelu (2010) found that males had larger heads then females at each of their sites, but each population also had unique growth rates^86^. Yet, sexual dimorphism in head length has been suggested as an ancestral morphological feature, with a genetic basis, and is present in both marine and freshwater populations^58^. However, our Procrustes LM found that sex and region significantly affected head shape, yet the effect size was larger for region (η^2^ = 0.169) and site nested into region (η^2^ = 0.464) compared to sex (η^2^ = 0.139) (Supplementary Table S4). Indicating that region accounted for more variance in head shape. However, this pattern was not apparent in the PCA plot for head shape (Fig. 3b). Unsurprising body morphology also differed significantly between sexes (Fig. 4a, Supplementary Table S5). Females were universally larger than males across habitats, with the greatest difference between average male and female body size found in tidal flat sites (Supplementary Table S5, Fig. S8). This larger morphological difference between male and female body size on tidal flats could be caused by the relatively large pelagic area in those habitats. For example, in their study of freshwater sticklebacks in Boulton Lake, BC, Reimchen and Nosil (2004) found that with more limnetic area in the lake, males and females occupied different niches. In this lake females were found in higher densities in the water column, while males lived in the benthic area, where they were likely guarding the nests^87^.

The body morphology PCA plot had a slightly more pronounced clustering pattern between males and females compared to head shape, but there was still some overlap on the PCA plot (Fig. 4a). This finding was similar to what Spoljaric and Reimchen (2008) observed among their six marine populations in northern Haida Gwaii, BC, where male and female clusters overlapped among marine populations, while freshwater sexes had distinct, well-separated clusters^44^. Our PCA plots found that females had larger pelvic girdles and more compressed heads and jaws compared to males, which compares to morphological variation seen by other researchers^44,58,59^. The Procrustes LM found that sex significantly affected body shape and had the largest effect size (η^2^ = 0.390) (Supplementary Table S6). While region (η^2^ = 0.210) and site nested in region (η^2^ = 0.132) also significantly affected body shape (Supplementary Table S6). Indicating that sex of the stickleback accounted for more variance in body shape, a pattern which was also evident in the PCA plot (Fig. 4).

### Juan de Fuca Strait population

The JdF site stood out in numerous ways in our study. It exhibited the opposite pattern to the other three regions, where female heads were larger than males and average JdF female and male head size differed by the smallest amount (Fig. 2). Pistore (2018) suggested that populations with lower dimorphism (smaller difference between male and female morphological characteristics) could be under stronger pressure from other drivers of morphology (e.g. food availability and predation), so they cannot expend extra energy on sexual dimorphism^88^. This JdF site area could in fact have unique predating intensity compared to other regions on average, as it is an area well protected from development.

This area also stood out in the PCA conducted on body shape (Fig. 6b, Supplementary Fig. S14). The Juan de Fuca Strait is tidally mixed. Tidal forces facilitate intense mixing of the brackish surface waters and the deep, saline waters. The other three oceanographic regions surrounding Vancouver Island have distinct water column stratification and are dominated by upwelling, downwelling, or Fraser River runoff^35^. This unique oceanographic region is likely contributing to the outlying morphological variation we observed, and we recommend future research to sample more marine populations in this region.

## Conclusion

This project was the first to look at multiple marine stickleback populations across the medium spatial scale of Vancouver Island and Southern mainland BC. We showed that these populations are variable. We expected to find some patterns of variation, such as between sexes. While other patterns of variation were unexpected, such as how head morphology did not follow the pattern of benthic-limnetic traits based on habitat availability and the unique sexually dimorphic patterns observed at the JdF site. It is clear that we cannot explain variation in marine sticklebacks the same way as for freshwater populations. Clearly, marine sticklebacks are deserving of a more focused study.

We have made several recommendations for future research throughout the text. Other recommendations would be to conduct a detailed study at a single location or estuary to disentangle factors that influence stickleback morphology on a smaller scale. As well as assessing reproductive status of sticklebacks, because marine stickleback exhibit ontogenetic changes in body and head shape^45^.

We should also note that some of the variation we observed in body size might be caused by differences in age or life span^6,89^, while body shape variability could be driven by differences in migration history (anadromous vs. sea-spawning marine populations) and the salinities encountered by each^29^. Anadromous sticklebacks likely pay a larger metabolic cost than marine sticklebacks due to their rapid shift in habitats, from freshwater to full-salinity open ocean, after the breeding season^74,90^. Therefore, a promising future venue of research would need to first decipher the links between age, migration, and otolith deposition in the lab.

The Canadian Pacific coast, in BC, has long been a hub for stickleback research in freshwater lakes and streams, coastal estuaries, marine bays and lagoons. Genetically and phenotypically divergent freshwater populations can be found throughout the Haida Gwaii archipelago^45,91^, Vancouver Island^92–94^, and Southern mainland BC^27,40,52^. Our study sets the stage for equivalent research programs to relate stickleback morphological variation to the marine scape they inhabit.

## Methods

Between May–July 2019, a total of 534 fish were collected from 15 sites around Vancouver Island and Southern BC, Canada (Fig. 1, Supplementary Table S2). At each site, sticklebacks were caught with beach seines at 1–2 m depth and 2–3 m offshore, or with un-baited minnow traps. Sticklebacks were collected and euthanized, through immersion in clove oil, following our animal use protocol approved by the University of Victoria Animal Care Committee, protocol number 2019–008, and were completed in accordance with the guidelines laid out by the ethics committee and the Department of Fisheries and Oceans (DFO) collection permits (XE-17-2019, XE-75-2019). Each fish was individually preserved in 95% ethanol. Three points of salinity and temperature were recorded at ~ 1 m depth at each site using a hand-held YSI (YSI Inc., Yellow Springs, OH, USA) during daylight hours. Sites were not revisited throughout the sampling season due to lack of time and the nature of our exploratory field season, and these salinity and temperature readings were not taken at similar points in the daily tidal cycle.

### Lab processing and fish imaging

In the lab, each fish was sexed by inspection of internal gonads, and fin clips were collected (stored in 95% ethanol) for archiving purposes. We excluded 14 specimens because they were either < 3 cm in length (thus likely juveniles), they had been severely injured with missing body parts, or because we could not properly identify the sex. The head of each specimen was photographed first using a SPOT Flex camera (SPOT Imaging, Diagnostic Instrument, Inc., Stirling Heights, MN, USA) mounted on a Wild Leica- M420 dissecting microscope at 10.5 × magnification (Leica Biosystems, Wetzlar, Germany). Each specimen was placed in a homemade apparatus to ensure each was photographed at the same position in the field of view. After completion of all headshots, the 534 specimens were photographed again to capture the body (referred to as body shots from here on). A Nikon D3500 (Nikon Corp., Tokyo, Japan) was mounted above the Styrofoam apparatus, thus each image was captured from 12 cm above a specimen. A ruler was placed in each photo as a scale bar for later calibration, and to extract head and body lengths from the photographs.

Stickleback lateral plates are heavily studied and an important morphological feature of the species. Thus we did visually assign a plate phenotype to each fish we processed. Each specimen was categorized into one of three plate morphs: low (10 or fewer plates with no keel), partial (11–25 plates, missing plates only on the middle of the body), or complete (26–35 plates)^1^. Yet, because of the small number of partial and low morphs, we combined the two into one category of non-complete plate morphs.

### Geometric morphometrics

**Figure 7:**
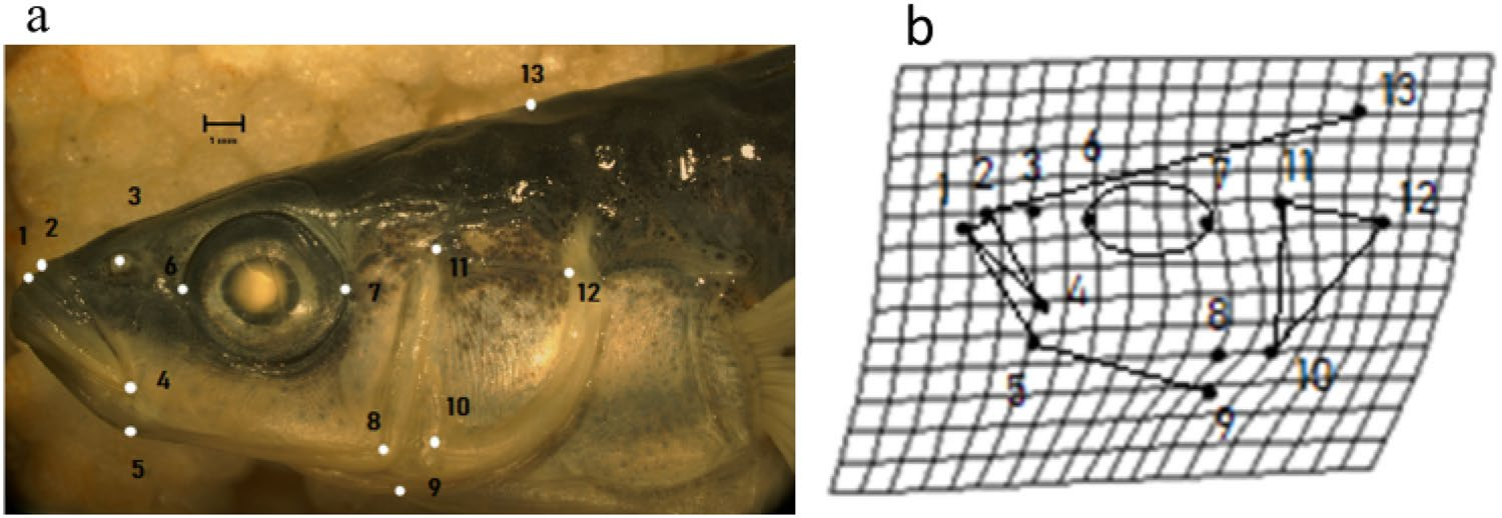
Analysis of stickleback head shape. Panel (**a**) shows an image of the left side of a female stickleback head sampled from the Black Creek Estuary site with anatomical landmarks (1–13) used for geometric morphometric analysis (see methods). Panel (**b**) is a thin-plate spline deformation grid which represents variation in the shape of the specimen in panel (**a**). The deformation grid is based on the Procrustes shape coordinates, generated from a generalized Procrustes analysis using the shape landmarks shows in panel (**a**) as described in the methods.

**Figure 8:**
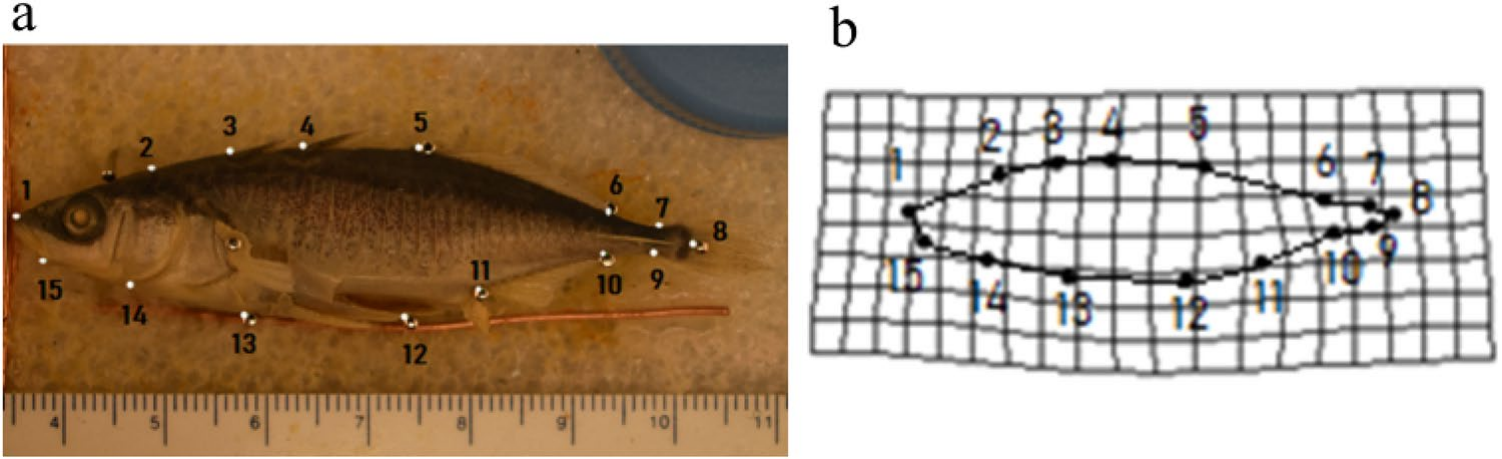
Analysis of stickleback body shape. Panel (**a**) shows an image of the left side of a female stickleback body sampled from the Englishman River site, with anatomical landmarks (1–15) used for geometric morphometric analysis (see methods). Panel (**b**) is a thin-plate spline deformation grid which represents variation in the shape of the specimen in panel (**a**). The deformation grid is based on the Procrustes shape coordinates, generated from a generalized Procrustes analysis using the shape landmarks shows in panel (**a**) as described in the methods.

The original images were converted into *tps* format using the software *tps.Util* version 1.61, and then organized into files for superimposition^95^. The *tps* images were uploaded into *tps.Dig* version 2.05 to digitize landmarks on the head and body shots^96^. For headshots, 13 anatomical landmarks were placed around the left side of the head (Fig. 7), which were adapted from landmarks used in previous studies^5,86^. For body shots, 15 landmarks were placed on the left side of the body (Fig. 8). The body landmarks were also based on previous studies^30,49^.

The landmarks used for geometric morphometric analysis of head shape were: 1. Anterior tip of the upper lip; 2. Anterior tips of the snout; 3. The nostril; 4. The axis of the jaws; 5. Posterioventral edge of angular; 6. Anterior edge of the eye; 7. Posterior edge of the eye; 8. Posterioventral edge of the third suborbital; 9. Anterioventral edge of the interoperculum; 10. Anterioventral edge of the operculum; 11. Anteriodorsal edge of the operculum; 12. Posteriodorsal edge of the operculum; 13. Posterior extant of the supraoccipital (Fig. 7). The bone description of landmarks was based on^24,86^.

The landmarks used for geometric morphometric analysis of body shape were: 1. Anterior tip of the upper lip; 2. Posterior extant of the supraoccipital; 3. Anterior base of the first dorsal spine; 4. Anterior base of the second dorsal spine; and 5. Anterior base of the third dorsal spine; 6. Posterior edge of the dorsal fin; 7. Anteriodorsal edge of the caudal fin; 8. Caudal end of the caudal keel; 9. Anterioventral edge of caudal fin base; 10. Posterior edge of ventral fin; 11. Anterior edge of anal fin; 12. Posterior process tip of the pelvic girdle; 13. Anterior process tip of the pelvic girdle; 14. Ventral tip of pectoral girdle; 15. Posterior edge of angular^59^ (Fig. 8).

### Generalized procrustes analysis

The head and body shape coordinates were analyzed separately. In the *geomorph* package, the “gpagen” function was used to perform a Generalized Procrustes Analysis (GPA)^97^. Following GPA, 26 shape variables were produced for the head dataset (X and Y coordinates for 13 landmarks), while 30 variables were produced for the body dataset (X and Y coordinates for 15 landmarks) (Supplementary Fig. S15, Fig. S16). Additionally, an extra vector was produced for each dataset which described the geometric size of each specimen’s head or body (i.e. CS)^98^.

### Linear mixed effects models of head and body size

Centroid size (CS) was highly correlated with head and body size (mm) (Supplementary Fig. S4, Fig. S9). Therefore, the analysis of head and body size was conducted on head length (mm) and standard length (cm) using linear mixed-effects models (LMMs) Linear mixed effect models were built using either head size (mm) or body size (cm) as the response variable. The predictor variables included in each global model were oceanographic region (region), coastal habitat type (habitat), sex, the interactions between sex and region, the interaction between sex and habitat. Site was included as a random effect. The global models were subjected to model selection based on corrected Akaike’s information criterion for small sample sizes (AICc)^99^. Following model selection, we decided not to average the top models (e.g. with ΔAICc < 4) because some of these models included interaction terms. We simply chose the model with the lowest AICc value for head and body size (as per recommendation of Scheipl et al*.* (2008)^100^).

All models were fitted with the *lme4* package for R, model selection was performed with the *MuMIn* package^101,102^. Multicollinearity was assessed between levels of each fixed effect using the “vif.mer” function for R^103^. Which calculated VIF values specifically for mixed-effect model fits with the *lme4* package. To further confirm the reliability of our top models, we checked for normality and homogeneity of the residuals across the regression line using histograms and qq-plots.

We preformed the regression of CS against head and body size (mm), which also showed a positive relationship between shape and size, or an allometric effect (Supplementary Fig. S4, Fig. S9). Additionally, we performed regressions among sexes and regions to observe the allometric effect by comparing regression slopes (Supplementary Fig. S5, Fig. S6, Fig. S10, Fig. S11).

### Principal components analysis

The “plotTangentSpace” function in the *geomorph* package for R performed PCA. To help visualize the shape differences, thin-plate spline deformation grids were generated using the “plotRefTarget” function^97^. Procrustes linear models (LMs) were built to test for differences in head and body shape (Procrustes shape variables) in relation to Log CS, sex, region, and habitat. Site was nested into oceanographic region as a random effect. Procrustes ANOVAs were performed using the “procD.lm” function in *geomorph* ^97^. Effect size (η^2^) was calculated as eta squared, or SS_effect_/SS_total,_ to further compare factors in each Procrustes linear model^104^.

We recognize that Canonical Variate Analysis (CVA) and Discriminant Function Analysis (DFA) can be helpful to find differences among groups, but it was our understanding that they are better utilized for data where all grouping structures are known. Since we did not have DNA analysis, we did not know the exact grouping structures of the data, and we decided it was more conservative and appropriate to use PCA. However, we did perform CVA analysis on the head and body data sets out of curiosity and it generally agreed with our findings, without providing new insight (Supplementary Fig. S17, S18, S19, S20).

## Supplementary Information

Supplementary Information.

## Data Availability

Data are available in the online data repository DataDryad.
