# Supplementary material for: Characterizing phenotypic diversity in marine populations of the threespine stickleback
Source: Sci Rep. 2022 Oct 26;12:17923. doi: 10.1038/s41598-022-22872-z (PMC9606258; doi:10.1038/s41598-022-22872-z)
Supplement: Supplementary file 1 — Supplementary Information. [file 41598_2022_22872_MOESM1_ESM.pdf]

Supplementary Information:
"Characterizing phenotypic diversity in marine populations of the threespine stickleback"
Authors: Ainsley L. Fraser and Rana El-Sabaawi

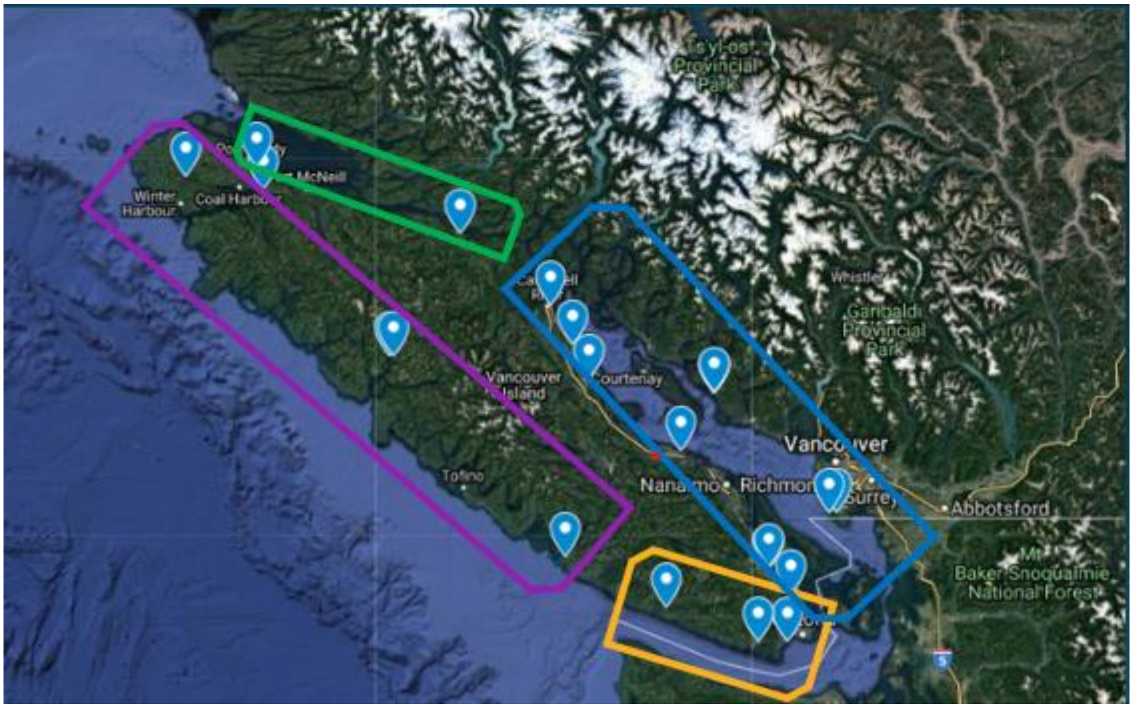

Figure S1. Map of the locations of our sites sampled on the Southern Mainland coast of BC and around Vancouver Island, BC. The Strait of Georgia is in the blue rectangle. The Juan de Fuca Strait is in the yellow rectangle and both Straits make up the Salish Sea. The western coast of Vancouver Island is in the purple, and the northern coast is in the green rectangle. Two points on this map are four separate sites (Moutcha Bay and the site on the Sunshine Coast). The sites were in close proximity on the map, they look like a single point on the map. All latitudinal and longitudinal coordinates can be found above in Table S1. Most sites can be accessed by paved road, but 4 sites were accessed along active logging roads (Bamfield South Inlet, Moutcha Bay (2 Sites), and Holberg, BC). Experience driving on logging roads is crucial. The base map was constructed using Google Earth (open access) at https://earth.google.com/web/.

Table S1. The average sea-surface temperatures (SST) and sea-surface salinities (SS Salinity) obtained from four BC lighthouses. Each lighthouse was chosen to represent average temperature and salinity values found in the four oceanographic regions around Vancouver Island, BC: Juan de Fuca Strait (JdF), the west coast (West), the North coast (North), and the Strait of Georgia (SoG). Reported are the average SST and SS salinities in May, June, and July each year. These datasets were collected, daily, from 1956-2019. Data was obtained from the Department of Fisheries and Oceans [61].

BC Lighthouse Name	Coordinates	Average SST (°C)			Average SS Salinity (PSU)			Region
		May	June	July	May	June	July	
Race Rocks	48.298, -123.532	9.84	10.6	11	31.2	31.1	31.1	JdF
Kains Island	50.441, -128.032	10.9	12.1	14.4	30.5	31.4	31.6	West
Amphitrite Point	48.922, -125.541	11.6	11.9	13.4	30	30.6	30.5	West
Nootka Point	49.593, -126.615	13.4	14.7	16.6	29.4	30.1	31.5	West
Pine Island	50.976, -127.728	9.46	9.7	11.3	31.4	31.5	31.5	North
Entrance Island	49.209, -123.808	12.8	15.7	16.7	27.5	24.9	25.2	SoG
Chrome Island	49.209, -124.685	12.9	15.2	17.4	28.3	26.8	26.6	SoG

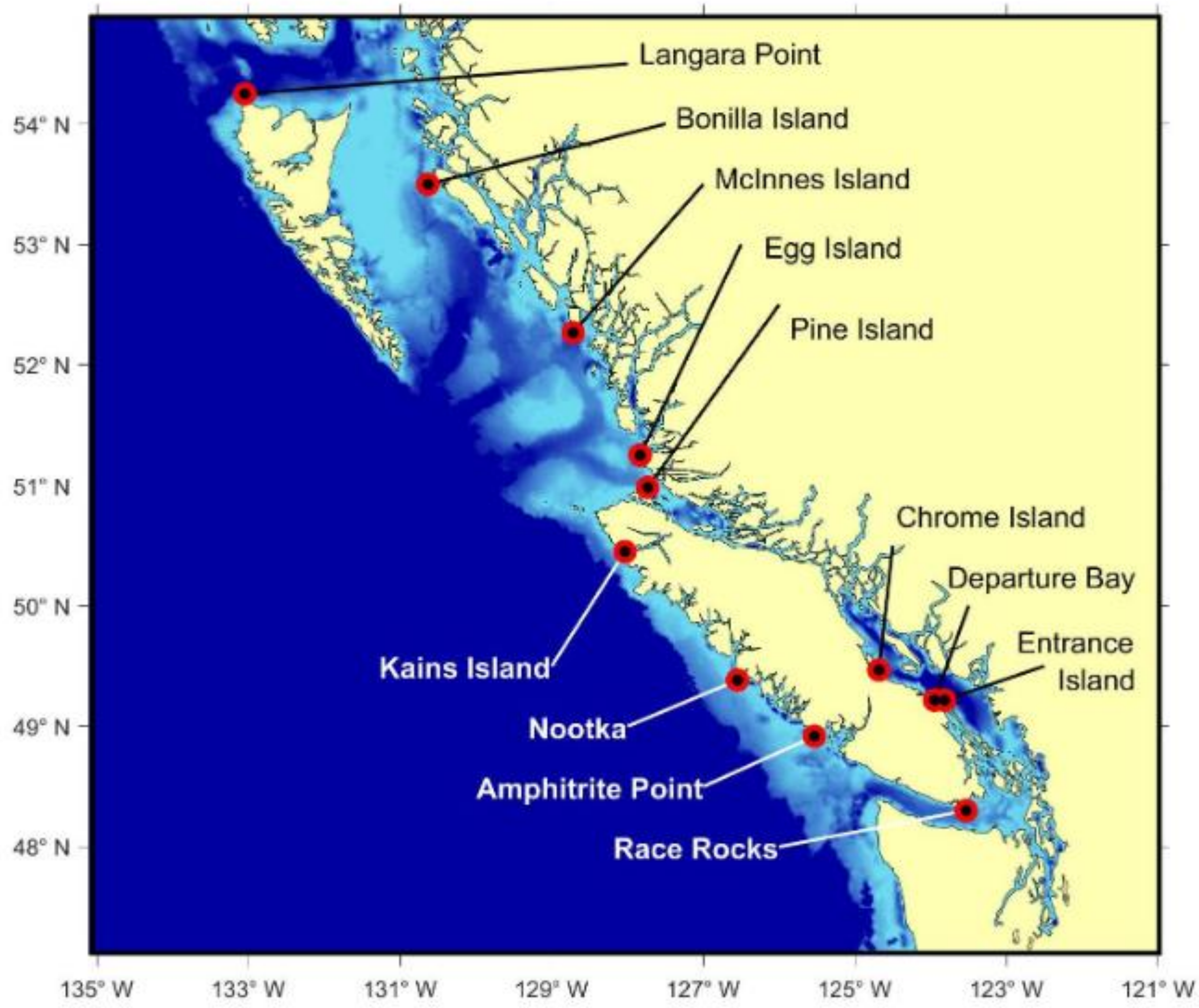

Figure S2. Map of BC lighthouse positions, including the lighthouses we reported in Table S2. The map shows the entire BC coastline, while the map in Figure S1 includes only Vancouver Island and Southern mainland BC. The image was constructed by the Department of Fisheries and Oceans (DFO) and downloaded from the data from BC lighthouses page (open access) at https://www.dfo-mpo.gc.ca/science/data-donnees/lightstations-phares/index-eng.html. Referenced from: Chandler, P.C., King, S.A., and Boldt, J. (Eds.). 2018. State of the physical, biological and selected fishery resources of Pacific Canadian marine ecosystems in 2017. Can. Tech. Rep. Fish. Aquat. Sci. 3266: viii + 245 p.

Table S2. List of sites including each respective site abbreviation, descriptions of where to park, where to deploy traps or seine net, GPS coordinates, date of collection during our 2019 field season, and total sticklebacks caught at each site with sex ratios between males (M) and females (F).

Location	Abbr.	Latitude & Longitude	Date of Collection	Description of Site	Fish Caught	
					M	F
Roberts Bank, off Tsawwassen, BC	RON	49.02945, -123.19034	12-May-19	Sampled with Raincoast Conservation Foundation. We collected by-catch from their team on a crab boat. Our contact was Dave Scott (dave@raincoast.org)	10	6
Englishman River Estuary	ENG	49.33161, -124.28803	15-May-19	Walk east around lagoon, and south along the stone fence. eelgrass beds present along the shore.	26	24
Courtenay River Estuary	COR	49.68108, -124.97257	18-May-19	From the Birdwatching Platform, walk out into Courtenay R. The deeper the better, best sampling at 1 hour after low tide.	6	7
Black Creek Estuary	BCE	49.85643, -125.10291	26-May-19	Place traps right in the creek. Park on opposite shore to the Provincial Park.	10	29
Oyster Lagoon	OYS	49.61232, -124.031	26-May-19	Park at these coordinates. Anywhere in the lagoon is good. Locals are familiar with stickleback research.	11	39
Salt Lagoon, Pender Harbour	STL	49.61446, -124.03043	26-May-19	Walk over from Oyster Lagoon. Very easy to catch fish anywhere and anytime of day.	31	19
Bamfield South Inlet	BAM	48.81331, -125.15667	03-Jun-19	Park in marked lot, walk down to the marked trail (200m), then follow side trail out to the flats. Place traps anywhere near vegetation on the flat.	22	15
Contuma River Estuary, Moutcha Bay	CMA	49.79257, -126.4445	08-Jun-19	We rented kayaks from Moutcha Bay Lodge Outfitters. Healthy eelgrass beds at the coordinates, left traps from low tide for 4 hours. Only access by boat/canoe/kayak.	21	8
Canton Lagoon, Moutcha Bay	CAN	49.79672, -126.47354	10-Jun-19	Can see lagoon from just off the logging road. Park in a truck pull out around the corner, walk along side of road. Left traps in shady areas during rising tide. Be aware of cougars.	15	15
Campbell River, BC	BAK	50.03921, -125.26754	20-Jun-19	Baike Island park. Would not recommend sampling here or anywhere in Campbell River, BC.	13	2
Salmon River, Sayward Estuary	SAY	50.37836, -125.94948	21-Jun-19	Cross active logging road, which runs parallel to the local road. There is parking for an estuary trail. Walk to the end of the estuary trail (600m) to a	36	6

				lagoon. Anywhere there is easy to catch stickleback.		
Holberg Estuary	HOL	50.65184, -128.00274	25-Jun-19	Very Rough logging road. Need a radio with "Holberg Rd. frequencies" from Western Forest Products North Island. Drive halfway down Dock Rd. On the inland side there are two pools which fill with the tide. Place traps here during rising tide.	5	8
Port Hardy Estuary	HDY	50.70001, -127.47179	27-Jun-19	At the end of Goodspeed Rd. park in Nature Trail parking. Walk across the bridge for bird viewing, and off to the left where there is a newly dug channel. Plenty of stickleback there.	19	33
Coles Bay Regional Park	COL	48.6298, -123.46732	03-Jul-19	Park in parking lot, walk down to the beach. Walk straight out and you will find large eelgrass beds. Seine anywhere there at low tide.	36	14
San Juan River	PRF	48.5665, -124.39376	09-Jul-19	Walk down flag-taped path at rising tide. Walk toward the River in the channel, place traps there.	11	37

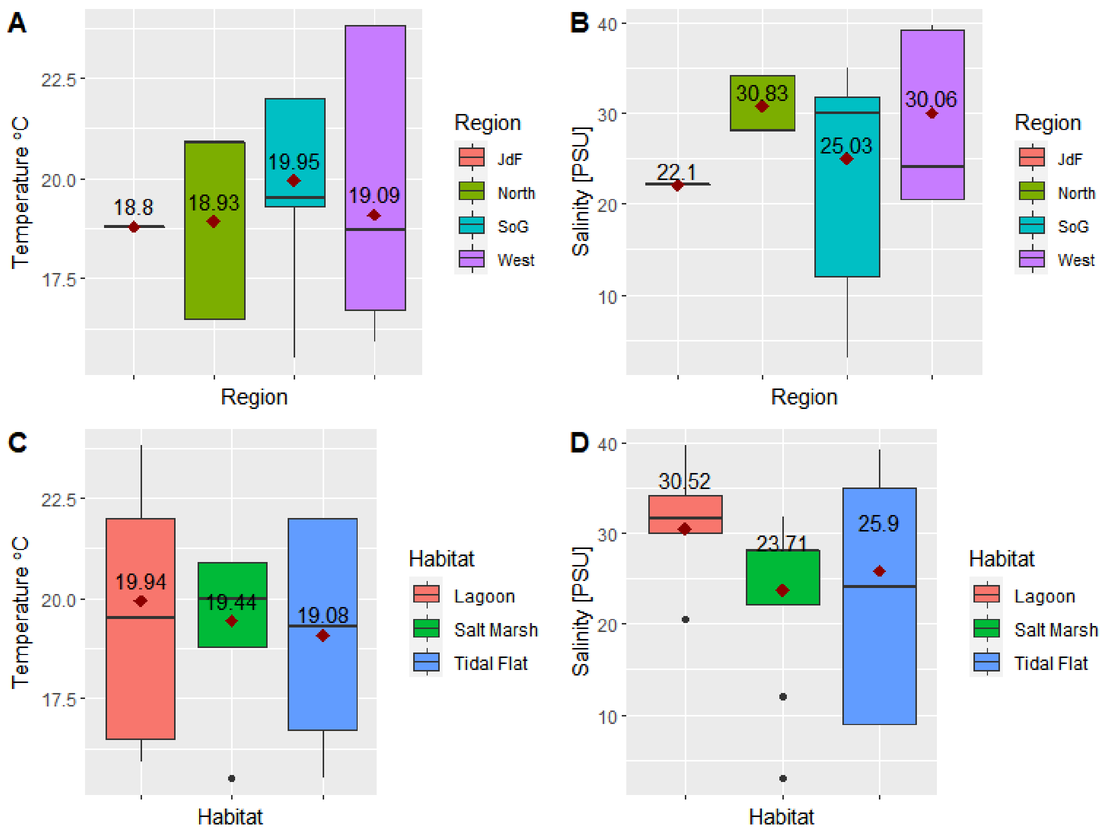

Figure S3. Box and whisker plots showing **[a]** regional variation in temperature (°C) sea surface temperatures (SST) at the time of sampling (spot samples) between the Juan de Fuca Strait (JdF), the Strat of Georgia (SoG), and the West and North coasts of Vancouver Island, and **[b]** regional variation in sea surface salinity (PSU), **[c]** habitat variation in SST, and **[d]** habitat variation in sea surface salinity (PSU). The data are averages of three sampling points collected during the survey.

Table S3. The best linear model explaining variation in head size (*i.e.* head length), based on model selection. Each fixed effect is separated by each level, excluding the reference category (The Strait of Georgia for region, females for sex). The estimate states if the level has a larger or smaller head size than the reference category of that fixed effect. The t-tests were calculated using Satterthwaite approximations to degrees of freedom. Collinearity was estimated between fixed effects in the global model based on VIF values (variance inflation factor). Also reported are standard error (SE), degrees of freedom (df), and the confidence interval (Conf. Interval). The response variable was specimen head size (mm), while sex, region, and the interaction between sex (Male [M] and Female [F]), and region (Rgn) were fixed effects. Site was included as a random effect (not shown). JdF is the Juan de Fuca Strait, SoG is the Strait of Georgia, North is the northern coast of Vancouver Island, and West is the west coast of Vancouver Island.

The linear mixed-effects model							
Fixed Effects	Estimates	SE	df	t-value	Conf. Interval	Pr (|> t|)	VIF
Intercept	13.55	0.316	12.2	42.8	12.93 – 14.17	**< 0.001**	-
Sex (M)	0.51	0.133	522	3.86	0.25 – 0.77	**< 0.001**	1.87
Rgn (JdF) $^{\Delta}$	-1.38	0.921	10.9	-1.50	(-3.19) – (0.42)	0.133	1.07
Rgn (North)	-1.35	0.702	11.9	-1.92	(-2.73) – (0.03)	0.054	1.17
Rgn (West)	-0.42	0.551	12.5	-0.766	(-1.50) – (0.66)	0.444	1.20
Rgn (JdF): Sex (M) $^{\Delta}$	-0.91	0.367	516	-2.48	(-1.63) – (-0.19)	**0.013**	1.17
Rgn (North): Sex (M)	0.02	0.280	523	0.0580	(-0.53) – (0.56)	0.954	1.38
Rgn (West): Sex (M)	0.46	0.243	519	1.91	(-0.01) – (0.94)	0.056	1.52

$^{\Delta}$The reference category was Rgn (SoG)

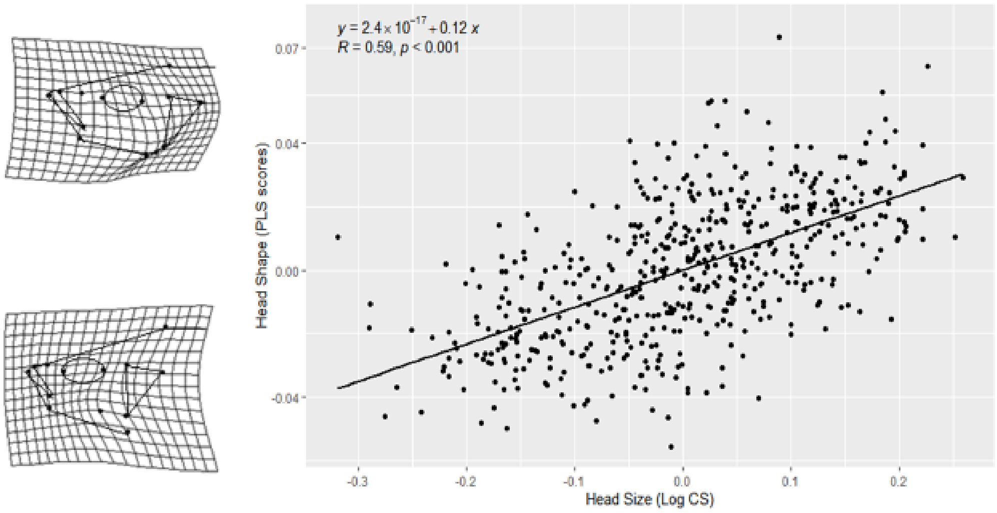

Figure S4. The relationship (r-PLS = 0.59, $p = 0.001$) between head shape and geometric head size (*i.e*. Log CS). Each symbol represents a single specimen. Side panels show the shape deformation grids based on between mean shape differences between the minimum (lower) and maximum (upper) Partial Least Squares (PLS) scores.

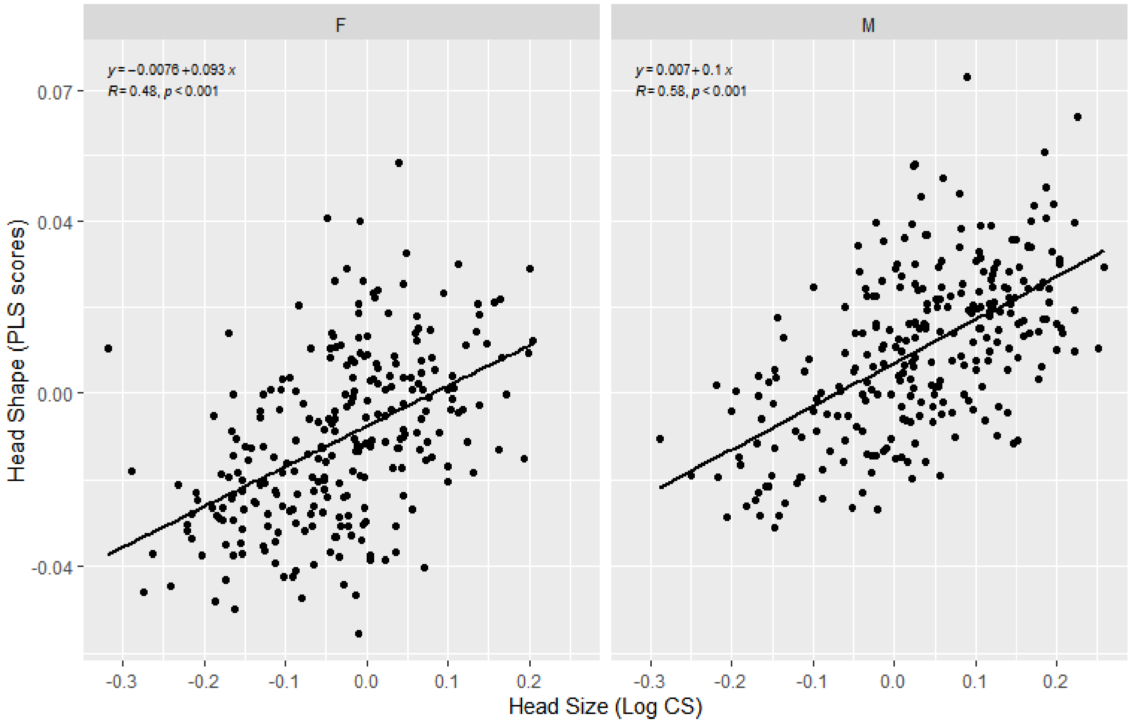

Figure S5. The relationship between head shape (*i.e.* Partial Least Squares scores) and geometric head size (*i.e.* Log CS) for males (M) and females (F). Each symbol represents a single specimen. The Pearson's correlation coefficient (*R*), subsequent p-values (*p*) and regression line equations were generated for each plot.

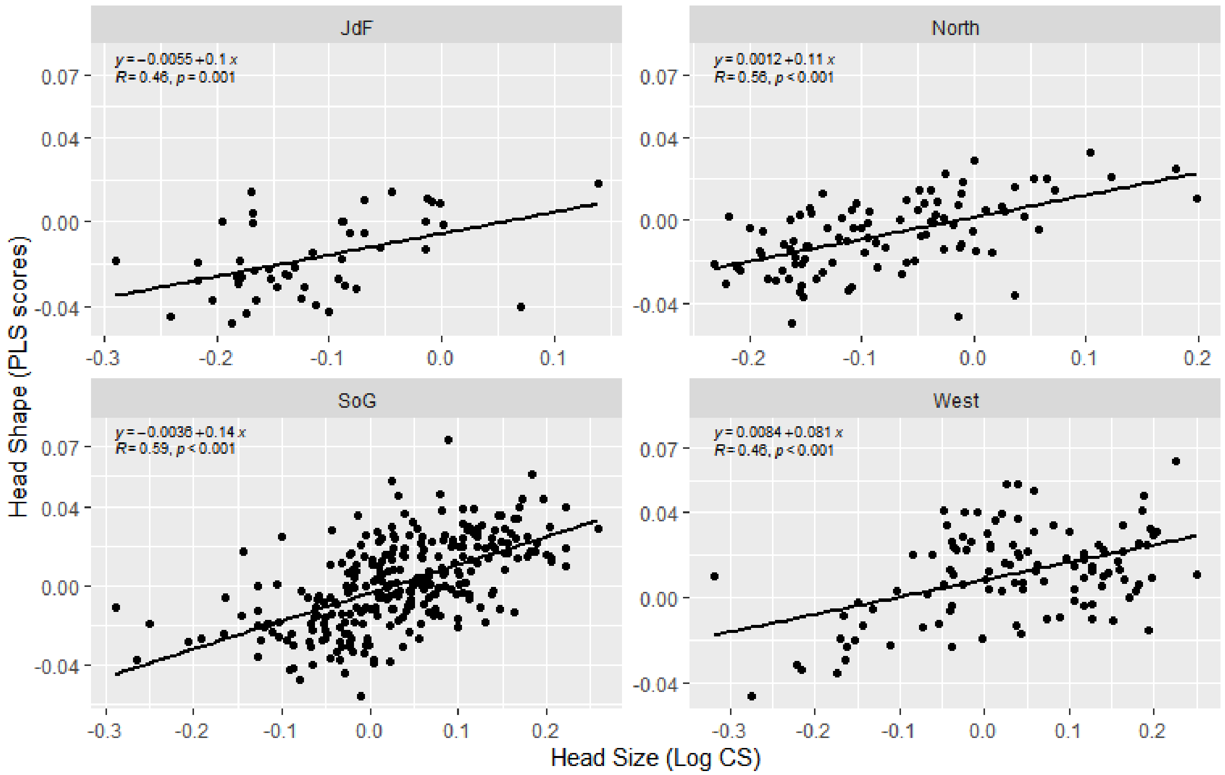

Figure S6. The relationship between head shape and geometric head size (*i.e.* Log CS) for each region: Juan de Fuca Strait (JdF), northern coast (North), Strait of Georgia (SoG) and western coast (West). Each symbol represents a single specimen. The Pearson's correlation coefficient (*R*), subsequent p-values (*p*) and regression line equations were generated for each plot.

Table S4. Results of the Procrustes linear model (LM) for head shape, which included log centroid size (Log CS), region (Rgn), sex, and the interaction between region and sex. Site was included as a nested random effect (not shown). Df = degrees of freedom, SS = sum of squares, η^2 = effect size (calculated as SS_{effect}/SS_{total}), MS= mean square, RSq= R-squared value, and F = F statistic.

Fixed Factors	Model Coefficients						
	df	SS	η^2	MS	RSq	F	p-value
Log CS	1	0.0891	0.196	0.0891	0.0449	29.900	0.001
Sex	1	0.0631	0.139	0.0631	0.0318	21.176	0.001
Rgn	3	0.0770	0.169	0.0257	0.388	8.609	0.001
Rgn : Sex	3	0.0142	0.0313	0.0192	0.00716	1.781	0.043
Rgn/Site	11	0.211	0.464	0.0192	0.106	6.432	0.001
Residuals	525	1.532	-	0.00298	0.771	-	-

Table S5. The best model explaining body size (*i.e.* standard length) variation, based on model selection. Each fixed effect is separated by each level, excluding the reference category (The Strait of Georgia for region, females for sex, and lagoons for habitat). The estimate states if the level has a larger or smaller body size than the reference category of that fixed effect. The t-tests were calculated using Satterthwaite approximations to degrees of freedom. Collinearity was estimated between fixed effects in the global model based on VIF values (variance inflation factor). Also reported are standard error (SE), degrees of freedom (df), and the confidence interval (Conf. Interval). The response variable was specimen body size (cm), while sex, habitat, region, the interaction between sex (Male [M] and Female [F]), and region (Rgn), and the interaction between sex and habitat (Hbt) were fixed effects. Site was included as a random effect (not shown). JdF is the Juan de Fuca Strait, SoG is the Strait of Georgia, North is the northern coast of Vancouver Island, and West is the west coast of Vancouver Island.

The linear mixed-effects model							
Fixed Factors	Estimates	SE	df	t-value	Conf. Interval	Pr (|> t|)	VIF
Intercept	5.58	0.171	9.65	25.4	5.15 – 6.01	**< 0.001**	-
Sex (M)	-0.259	0.07	517	-3.4	(-0.41) – (-0.11)	**0.001**	3.92
Rgn (JdF) $^{\Delta}$	-1.13	0.344	9.23	-2.54	(-2.0) – (-0.26)	**0.011**	1.26
Rgn (North)	-0.775	0.256	9.94	-2.38	(-1.41) – (-0.14)	**0.017**	1.29
Rgn (West)	-0.161	0.205	9.86	-0.61	(-0.67) – 0.35	0.54	1.36
Hbt (Salt Marsh) *****	0.323	0.225	10	1.14	(-0.23) – 0.89	0.25	1.87
Hbt (Tidal Flat)	0.648	0.202	9.73	2.51	0.14 – 1.15	**0.012**	1.52
Hbt (Salt Marsh): Sex (M)	-0.0476	0.116	520	-0.406	(-0.28) – 0.18	0.685	2.84
Hbt (Tidal Flat): Sex (M)	-0.235	0.094	515	-2.49	(-0.42) – (-0.05)	**0.013**	2.34
Rgn (JdF): Sex (M)	-0.122	0.172	517	-0.701	(-0.46) – 0.22	0.483	1.55
Rgn (North): Sex (M)	0.174	0.125	520	1.37	(-0.07) – 0.42	0.170	1.76
Rgn (West): Sex (M)	0.196	0.0988	516	1.97	0.00 – 0.39	0.049	1.61

$^{\Delta}$ The reference category was Rgn (SoG)
*The reference category was Hbt (Lagoon)

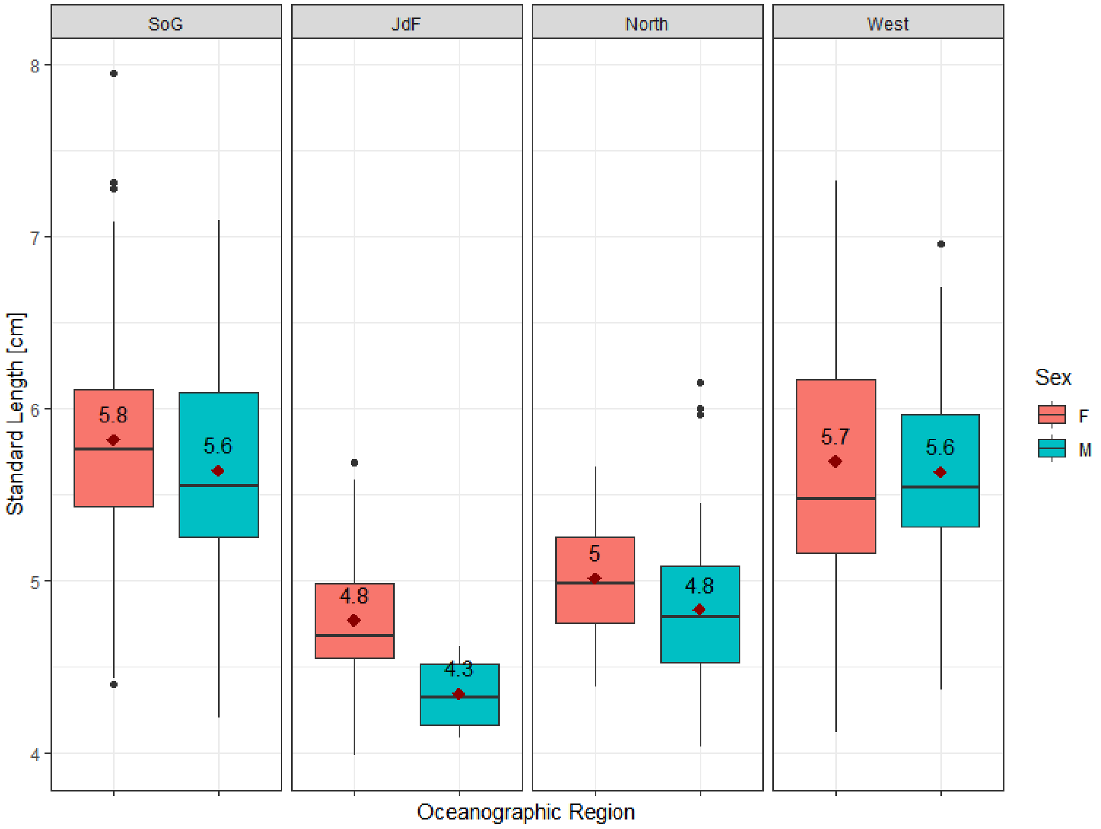

Figure S7. Differences between male and female body size, represented by standard length (cm), observed in each oceanographic region. The regions include the Juan de Fuca Strait (JdF), the northern coast of Vancouver Island (North), Strait of Georgia (SoG), and the west coast of Vancouver Island (West). The solid black lines represent the median standard length, while the red triangles represent the average standard length for each region (with the average value in black above each point).

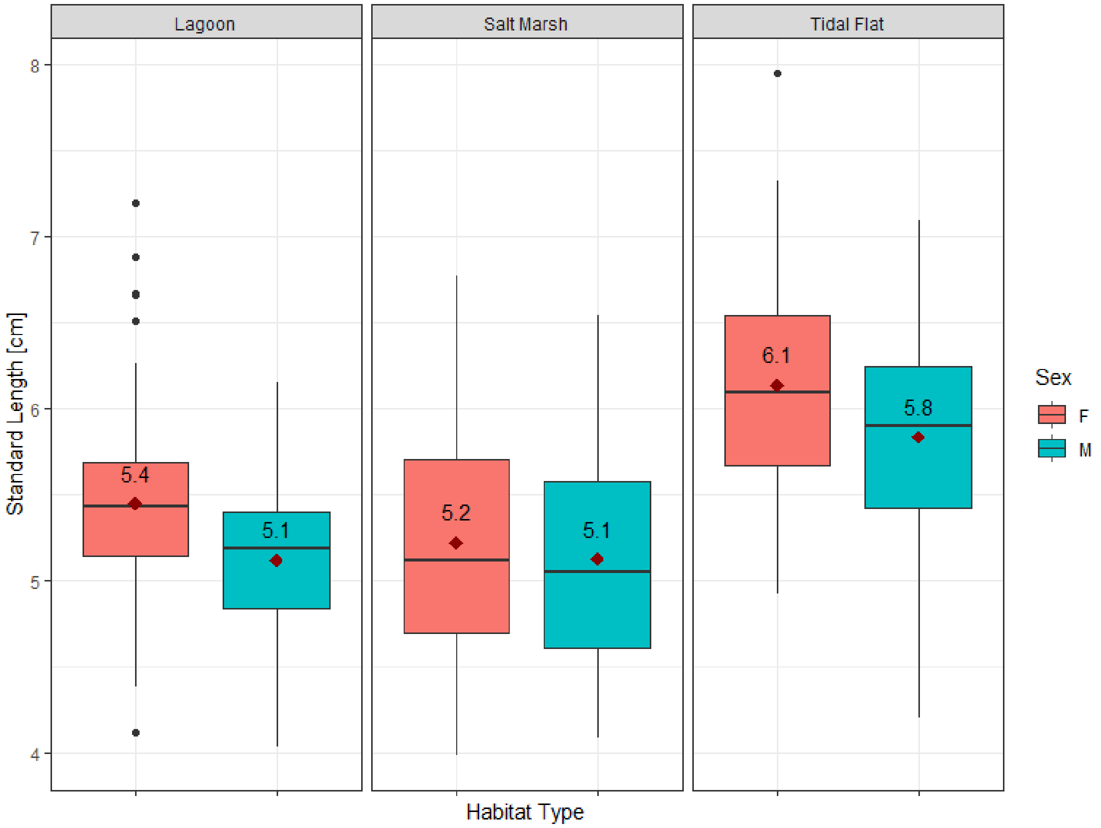

Figure S8. Sexual dimorphism in body size, represented by standard length (cm), observed among different habitat types. Males (M) and females (F) are shown in each habitat type. The solid black lines represent the median standard length. The red triangles represent the average standard length (with the average value in black above each point).

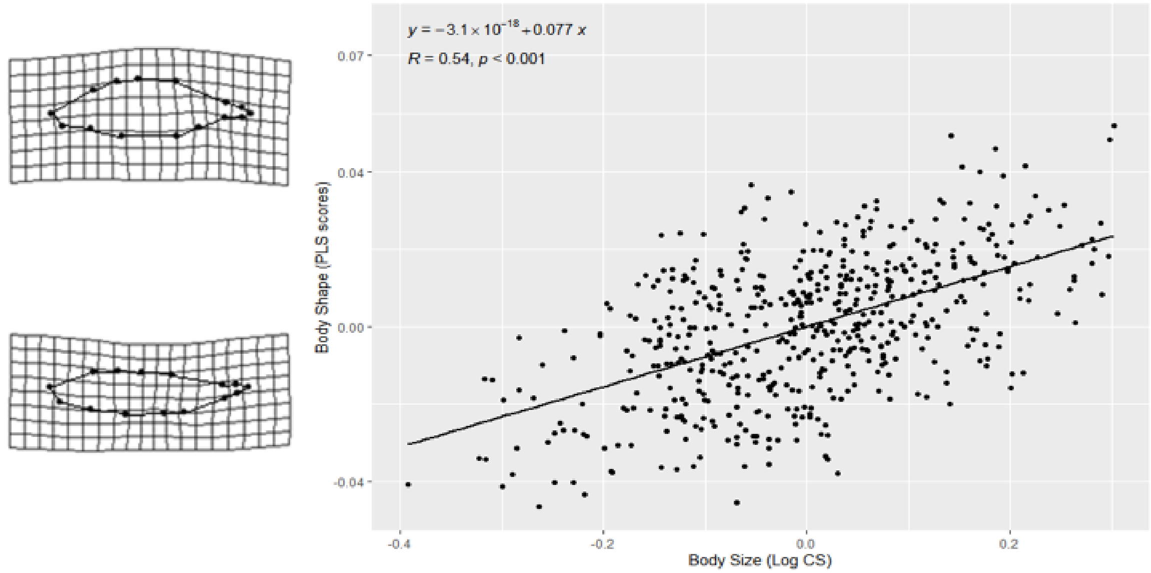

Figure S9. The relationship (r-PLS = 0.54, p = 0.001) between body shape and geometric body size (*i.e*. Log CS). Each symbol represents a single specimen. Side panels show the shape deformation grids based on between mean shape differences between minimum (lower) and maximum (upper) Partial Least Squares (PLS) scores.

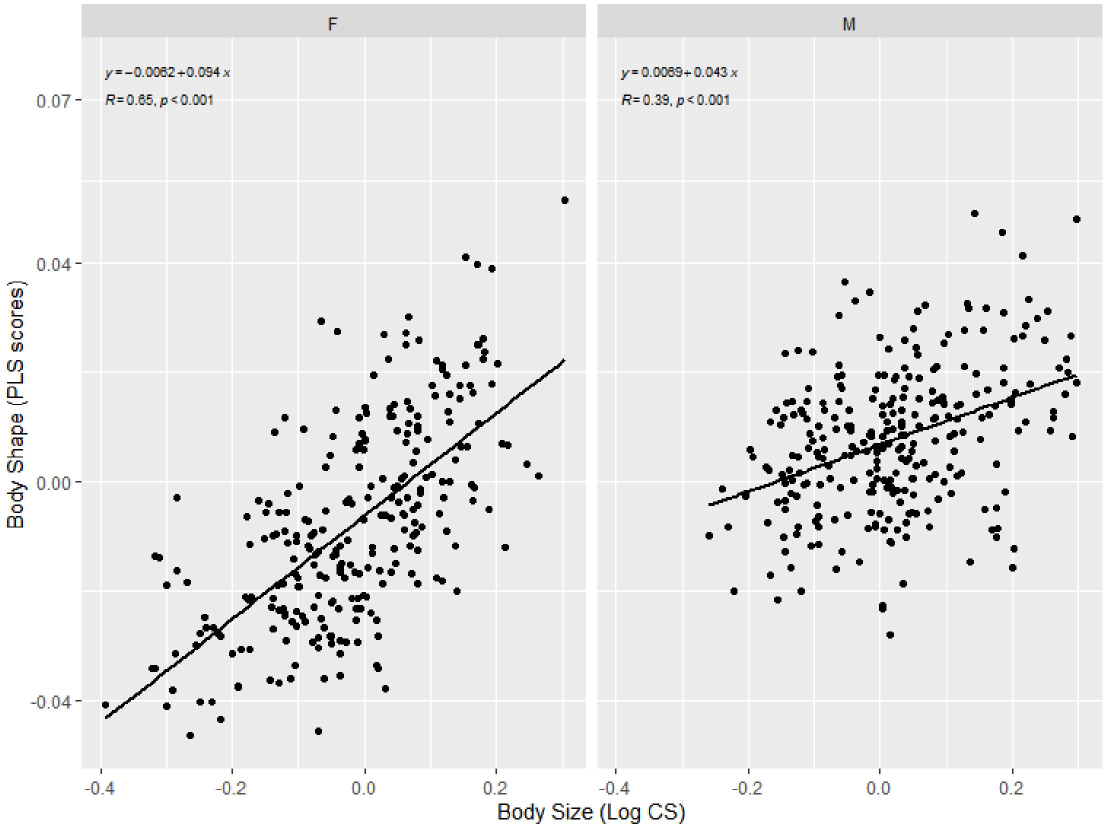

Figure S10. The relationship between body shape and geometric body size (*i.e*. Log CS) for males (M) and females (F). Each symbol represents a single specimen. The Pearson's correlation coefficient (*R*), subsequent p-values (*p*) and regression line equations were generated for each plot.

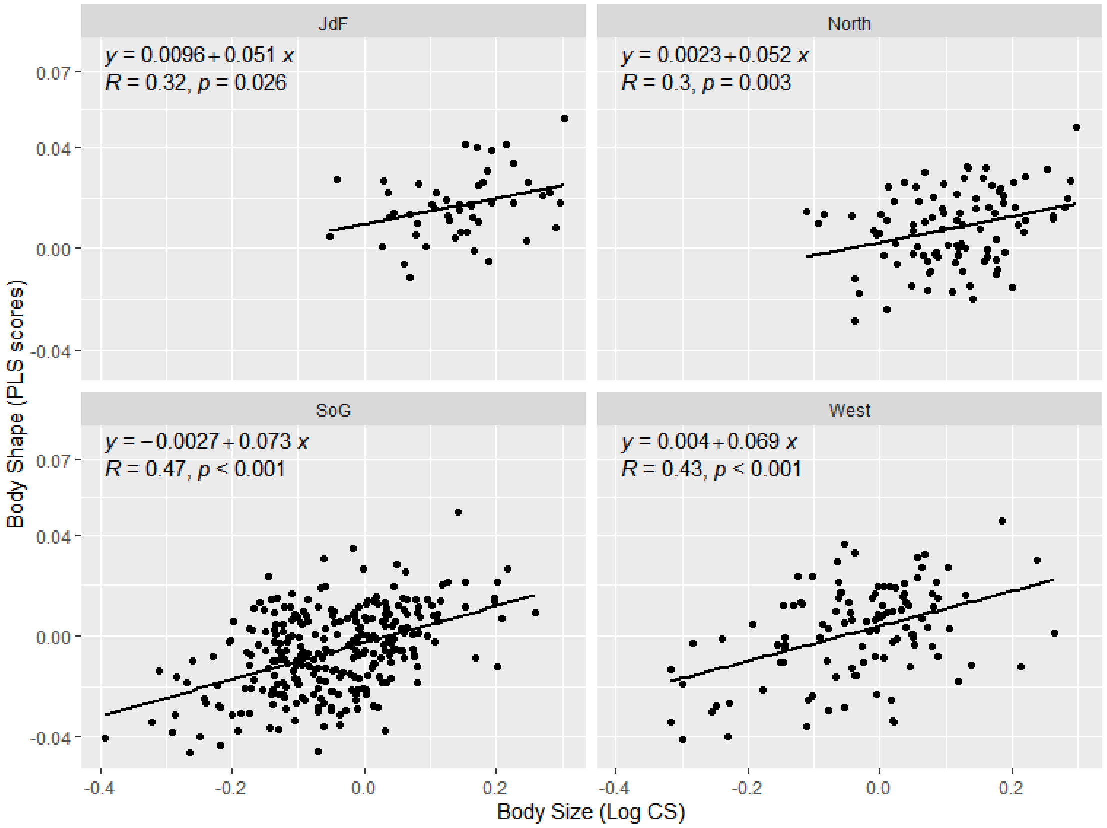

Figure S11. The relationship between body shape and geometric body size (*i.e.* Log CS) for each region: Juan de Fuca Strait (JdF), northern coast (North), Strait of Georgia (SoG) and western coast (West). Each symbol represents a single specimen. The Pearson's correlation coefficient (*R*), subsequent p-value (*p*) and regression line equation were generated for each plot.

Table S6. Results of the Procrustes linear model (LM) for body shape, which included log centroid size (Log CS), region (Rgn), habitat (Hbt), and sex. Site was included as a nested random effect (not shown). Df = degrees of freedom, SS = sum of squares, η^2 = effect size (calculated as SS_{effect}/SS_{total}), MS = mean square, RSq = R-squared value, and F = F statistic.

Factors	Model Coefficients						
	df	SS	η^2	MS	RSq	F	p-value
Log CS	1	0.051	0.153	0.51	0.0487	36.8	0.001
Sex	1	0.13	0.390	0.13	0.128	96.9	0.001
Rgn	3	0.070	0.210	0.023	0.0666	16.8	0.001
Hbt	2	0.019	0.0570	0.0097	0.0185	7.00	0.001
Sex : Hbt	3	0.0075	0.0225	0.0038	0.0072	2.73	0.001
Sex : Rgn	2	0.012	0.0360	0.0040	0.0115	2.90	0.001
Rgn/Site	9	0.044	0.132	0.0048	0.420	3.50	0.001
Residuals	517	0.72	-	0.001	0.695	-	-

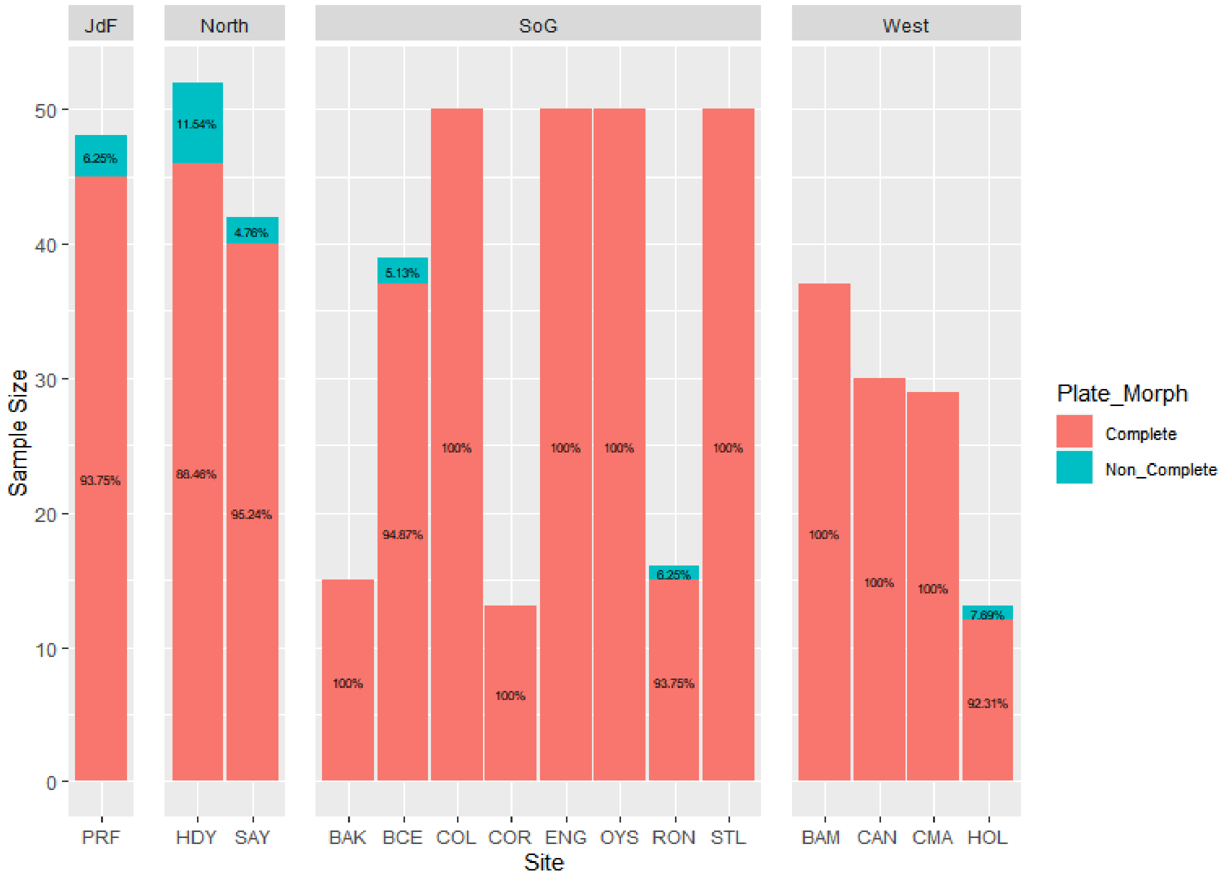

Figure S12. The proportion of complete plate morphs represented by the red portion of the bar vs. non-complete plate morphs in blue (*i.e.* low and partial plate morphs) at each site. Each sampling site is separated into the respective oceanographic region, Juan de Fuca Strait (JdF), Strait of Georgia (SoG), the west and northern coasts of Vancouver Island, BC. Sample size from each site on the y-axis. Percentage values in each bar indicate the total proportion value for that individual site. Site abbreviations are identified in Table S1.

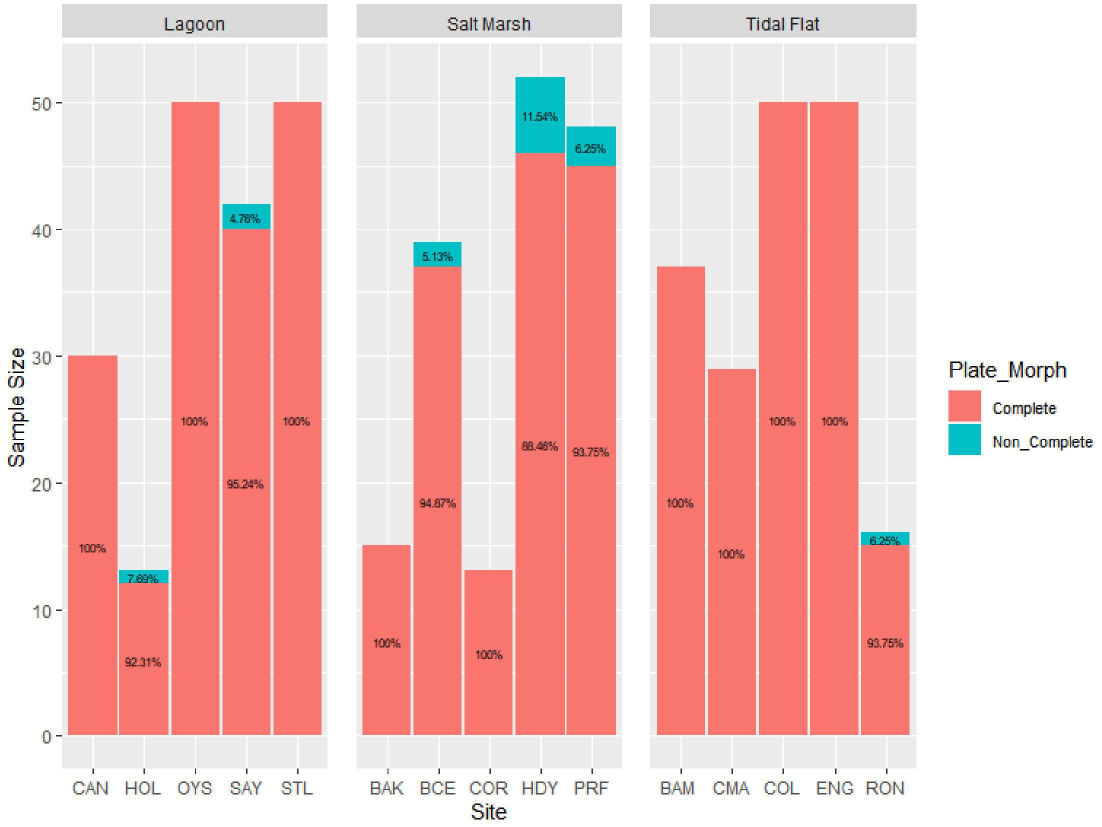

Figure S13. The proportion of complete plate morphs as the red portion of the bar vs. non-complete plate morphs in blue (*i.e.* low and partial plate morphs) at each sampling site. Each site is also separated into the respective habitat type for each site, with total sample size from each site on the y-axis. Percentage values in each bar indicate the total proportion value for that individual site. Site abbreviations are identified in Table S1.

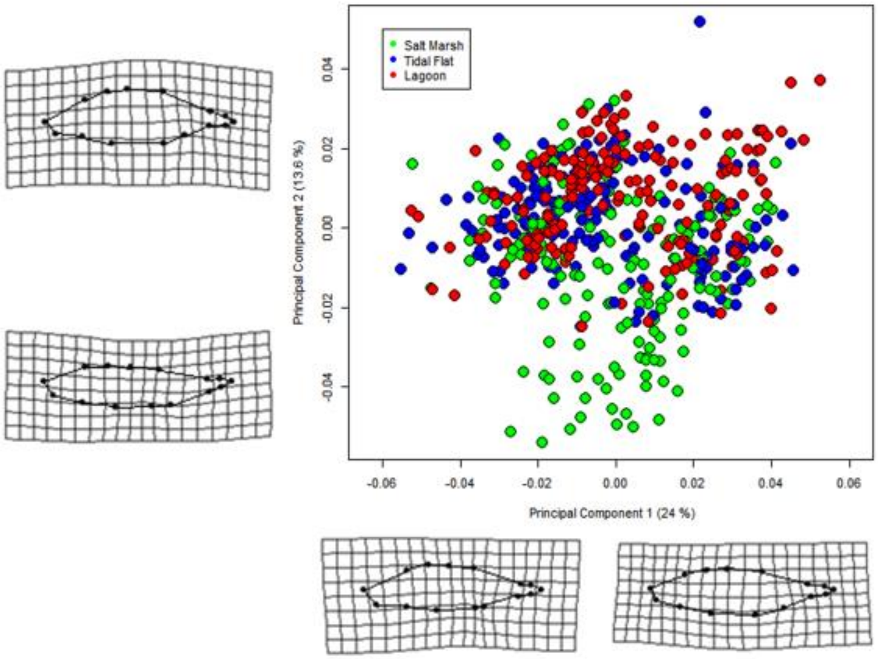

Figure S14. Principal Component Analysis (PCA) of body shape. The body shape for each specimen was obtained from a set of fifteen anatomical landmarks (Fig. 7). The landmarks were converted into 30 shape variables by way of a Generalized Procrustes Analysis (GPA). These vectors of shape were used to carry out the PCA. Each point represents data from a specimen sampled from a habitat type, which include salt marshes (light green), tidal flats (dark blue), and lagoons (red). The body shape variation is illustrated with the four deformation grids, which represent the differences between the specimen on each end of a Principal Component (PC) axis (PC1 and PC2).

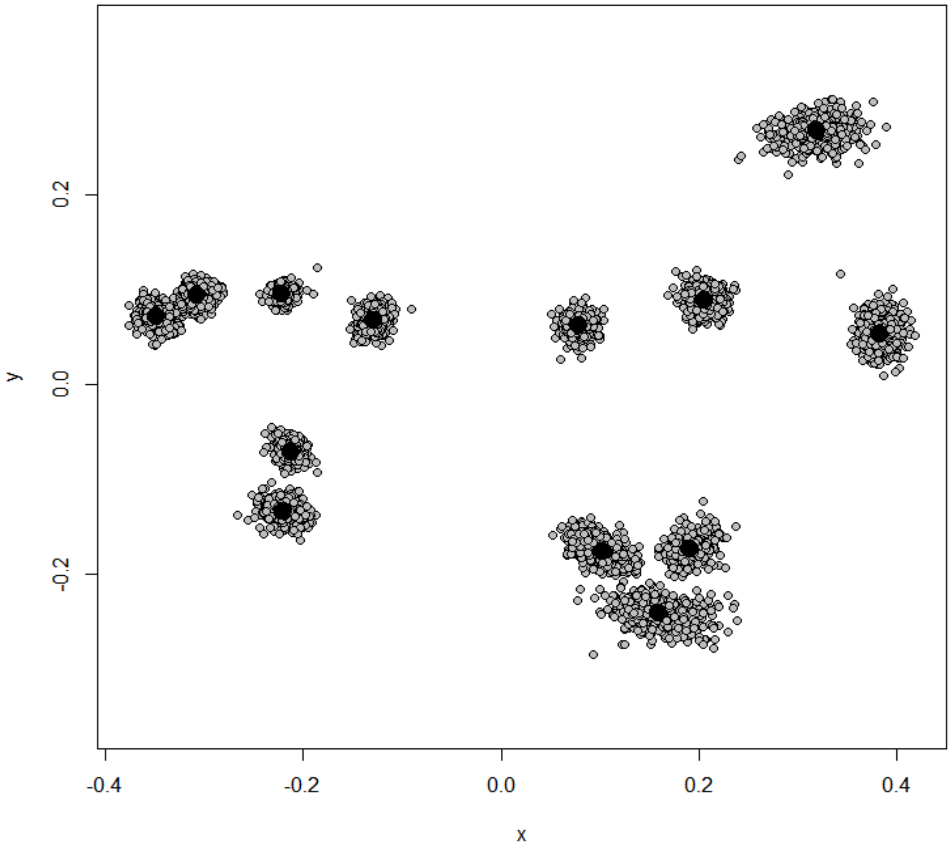

Figure S15. All landmarks of individuals (*i.e.* Procrustes shape variables) superimposed on top of one another after Generalized Procrustes Analysis (GPA) of head shots. The snout of the individual is the left-most landmark, while the top of the head is the upper right-most landmark.

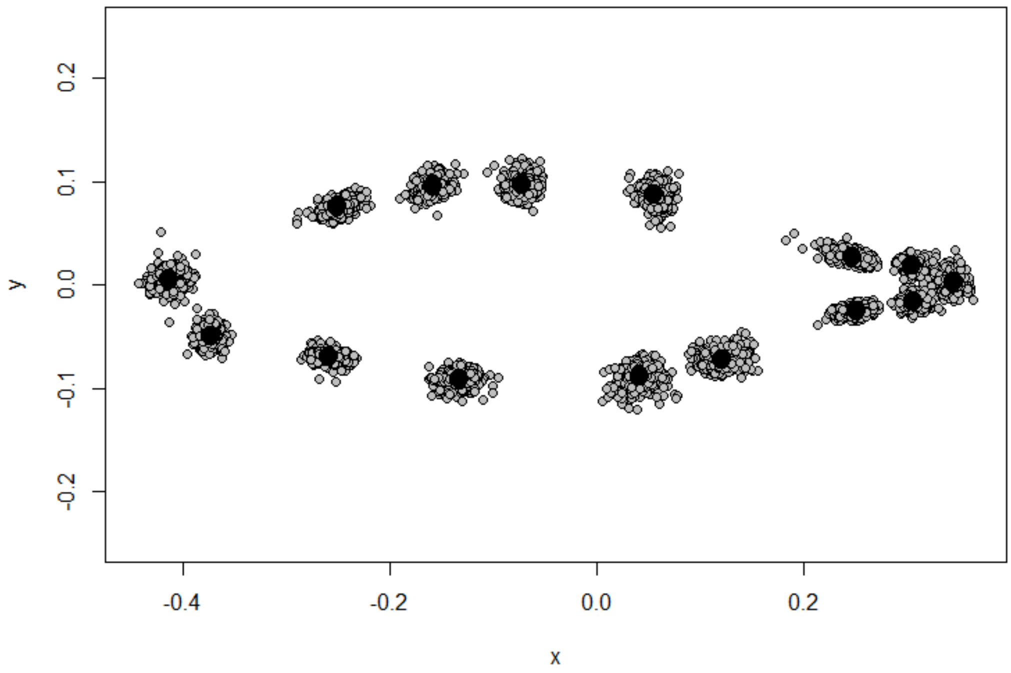

Figure S16. All landmarks of individuals (*i.e.* Procrustes shape variables) superimposed on top of one another after Generalized Procrustes Analysis (GPA) of body shots. The snout of the individual is the left-most landmark, while the caudal fin is the right-most landmark.

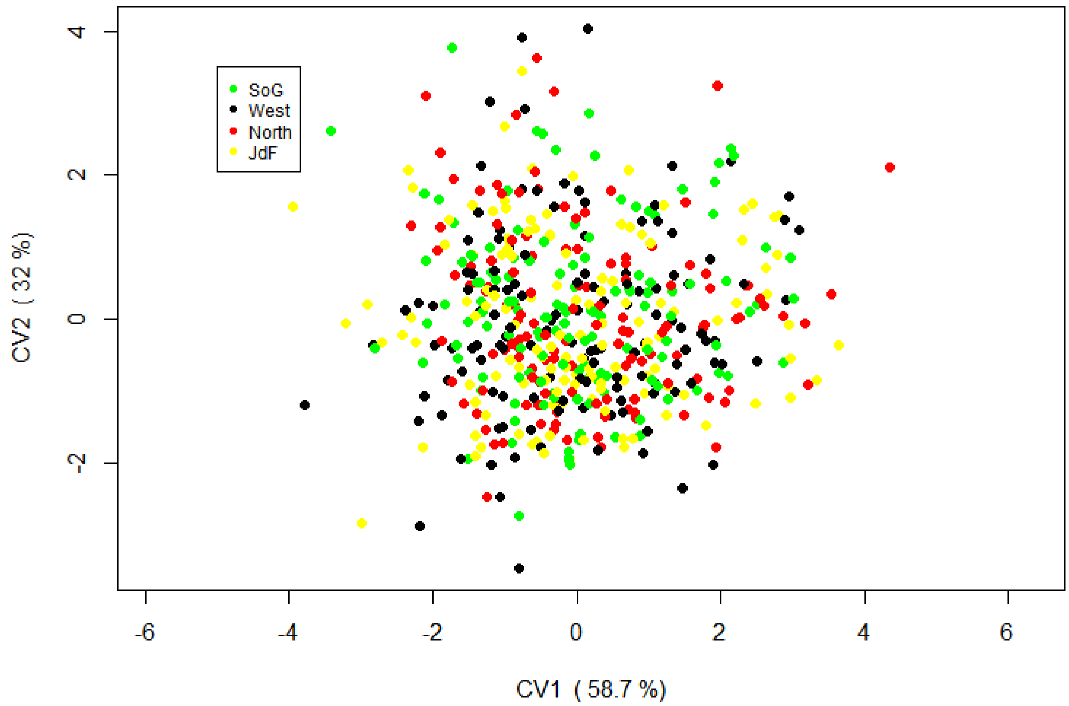

Figure S17. Canonical Variate Analysis (CVA) of head shape. The landmarks were converted into 26 shape variables by way of a Generalized Procrustes Analysis (GPA). These vectors of shape were used to carry out the CVA. Each data point represents data from the four oceanographic regions that surround Vancouver Island, BC: Strait of Georgia (green), west coast Vancouver Island (black), north coast Vancouver Island (red), and the Juan de Fuca Strait (yellow). Canonical Variate 1 (CV1) axis represents 58.7% of the head shape variation and CV2 represents 32.0% of the head shape variation.

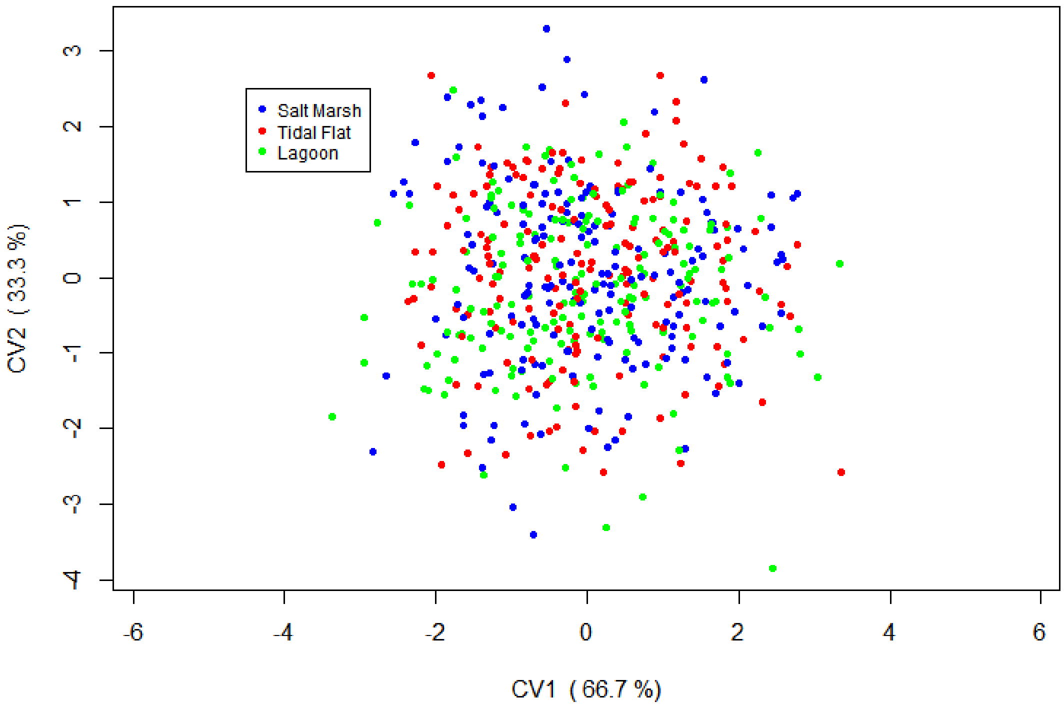

Figure S18. Canonical Variate Analysis (CVA) of head shape. The landmarks were converted into 26 shape variables by way of a Generalized Procrustes Analysis (GPA). These vectors of shape were used to carry out the CVA. Each point represents data from a specimen sampled from a habitat type, which include salt marshes (light green), tidal flats (dark blue), and lagoons (red). Canonical Variate 1 (CV1) axis represents 66.7% of the head shape variation and CV2 represents 33.3% of the head shape variation.

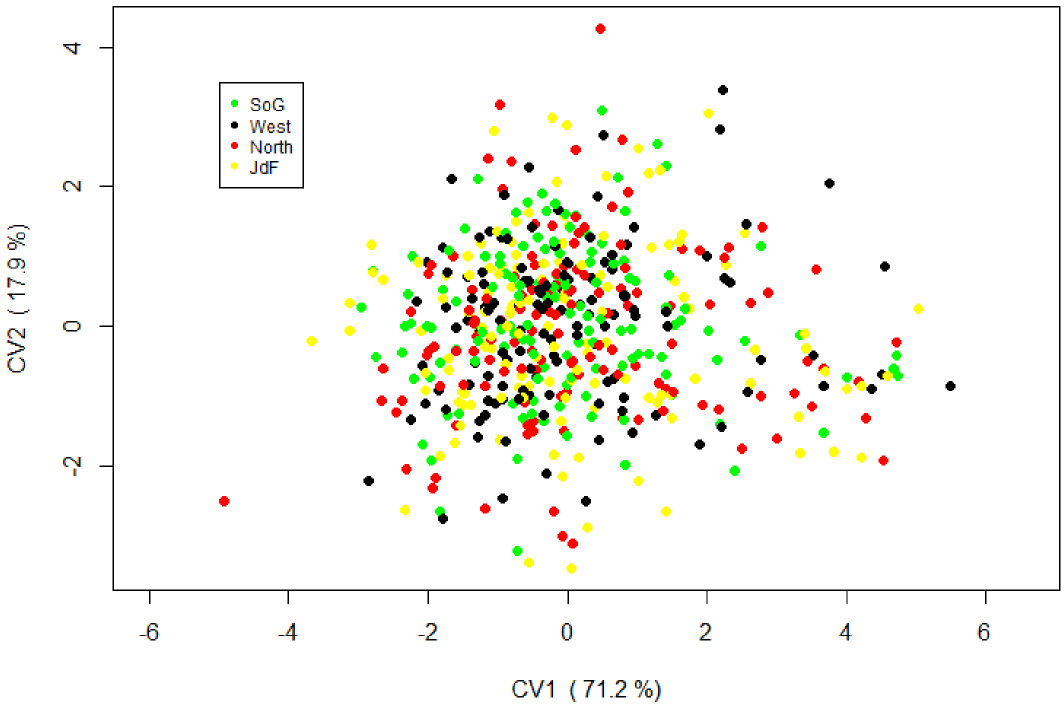

Figure S19. Canonical Variate Analysis (CVA) of body shape. The landmarks were converted into 30 shape variables by way of a Generalized Procrustes Analysis (GPA). These vectors of shape were used to carry out the CVA. Each data point represents data from the four oceanographic regions that surround Vancouver Island, BC: Strait of Georgia (green), west coast Vancouver Island (black), north coast Vancouver Island (red), and the Juan de Fuca Strait (yellow). Canonical Variate 1 (CV1) axis represents 71.2% of the body shape variation and CV2 represents 17.9% of the body shape variation.

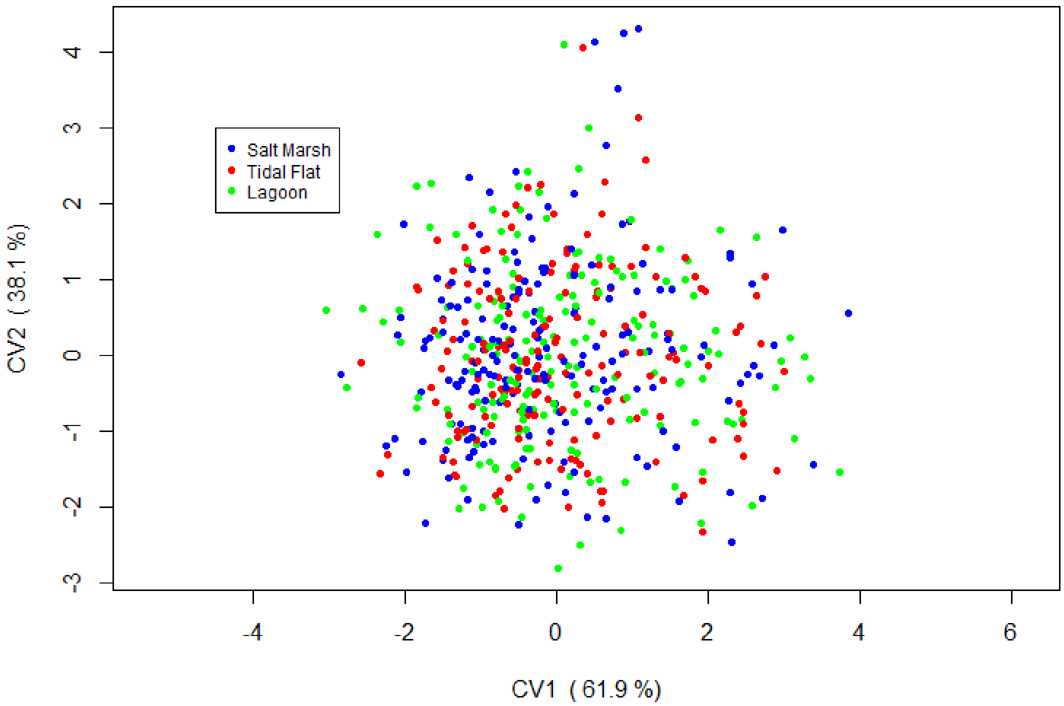

Figure S20. Canonical Variate Analysis (CVA) of head shape. The landmarks were converted into 30 shape variables by way of a Generalized Procrustes Analysis (GPA). These vectors of shape were used to carry out the CVA. Each point represents data from a specimen sampled from a habitat type, which include salt marshes (light green), tidal flats (dark blue), and lagoons (red). Canonical Variate 1 (CV1) axis represents 61.9% of the body shape variation and CV2 represents 38.1% of the body shape variation.
